# Nanomaterial-Based Autophagy Modulation: Multiple Weapons to Inflame Immune Systems and the Tumor Microenvironment

**DOI:** 10.34133/bmr.0111

**Published:** 2025-04-14

**Authors:** Min Jiang, Xinyi Zhang, Zhilei Cui, Meng Li, Huifen Qiang, Keqin Ji, Meigui Li, Xinyang Xuan Yuan, Beibei Wen, Qian Xue, Jie Gao, Zhengmao Lu, Yan Wu

**Affiliations:** ^1^ Department of Gastrointestinal Surgery, The First Affiliated Hospital of Naval Medical University, Shanghai 200433, China.; ^2^ College of Life Science, Mudanjiang Medical University, Mudanjiang 157011, China.; ^3^ Changhai Clinical Research Unit, The First Affiliated Hospital of Naval Medical University, Shanghai 200433, China.; ^4^ Department of Respiratory Medicine, XinHua Hospital Affiliated to Shanghai Jiao Tong University School of Medicine, Shanghai 200092, China.; ^5^ Department of Dermatology, Shanghai Children’s Medical Center, Shanghai Jiaotong University School of Medicine, Shanghai 200127, China.; ^6^ School of Pharmacy, Henan University, Kaifeng 475004, China.; ^7^ Department of Dermatology, The First Affiliated Hospital of Naval Medical University, Shanghai 200433, China.; ^8^ Shanghai Key Laboratory of Nautical Medicine and Translation of Drugs and Medical Devices, Shanghai 200433, China.

## Abstract

Autophagy, a fundamental cellular process, is a sensitive indicator of environmental shifts and is crucial for the clearance of cellular debris, the remodeling of cellular architecture, and the facilitation of cell growth and development. The interplay between stromal, tumor, and immune cells within the tumor microenvironment is intricately linked to autophagy. Therefore, the modulation of autophagy in these cell types is essential for developing effective cancer treatment strategies. This review describes the design and optimization of nanomaterials that modulate autophagy in tumor-associated and immune cells. This review elucidates the primary mechanisms by which nanomaterials induce autophagy and discusses their application in cancer therapy, underscoring the potential of these materials to eradicate cancer cells, bolster the immune response, and elicit robust, enduring antitumor immunity, thereby advancing the frontiers of oncological treatment.

## Introduction

Relevant data show that in 2020, 61% of global deaths were caused by noncommunicable diseases (NCDs), with cancer being the second leading cause of NCDs [1]. In recent years, cancer incidence and mortality rates have continued to rise worldwide, making cancer one of the most likely contributors to human mortality worldwide, and it has been defined as the greatest health problem facing the world’s human population [2]. According to published data for 2020, approximately 19.3 million individuals were newly diagnosed with cancer, while the number of deaths due to cancer was approximately 10 million. Researchers calculate that by around 2040, 28.4 million people are expected to have cancer, 147% of the number known in 2020 [1]. With the increase in cancer incidence and mortality rates, relevant research on cancer treatment has gradually increased, and the relative survival rate of cancer patients has improved to some extent. Currently, the most commonly applied cancer treatments are surgical resection, radiotherapy, chemotherapy, immunotherapy, etc. However, most treatment options are limited by insufficient drug utilization, susceptibility to drug resistance, and high cytotoxicity [3]. As a result, all oncology researchers are confronted with the same issue: minimizing the adverse effects of cancer treatment, achieving greater effects of tumor treatment in the shortest amount of time, and realizing precision tumor treatment.

Autophagy is a conserved evolutionary process that can effectively prevent cellular damage, promote the continued survival of cells in the absence of nutrients, and respond to cytotoxic stimuli and is often regarded as a pro-cell survival mechanism [4]. From De Duve’s discovery of lysosomes in 1969 to Yoshimori’s discovery of autophagy-related mechanisms in 2016, 2 Nobel Prizes have made autophagy one of the most complex processes in the development of cell survival [5,6]. Previously, autophagy was considered a general form of programmed cell death distinct from apoptosis, but as research on the mechanisms of autophagy has progressed, the definition of autophagy has gradually shifted to a broader process of stress-resistant cell degradation. When autophagy is triggered in healthy cells, it often has antiapoptotic effects; however, when autophagy is triggered in diseased cells, it usually results in cell death or triggers apoptosis [7]. Furthermore, cancer cells and other cells in the tumor microenvironment (TME) can undergo autophagy in response to a combination of intra- and external stress signals, such as metabolic stress, hypoxia, redox stress, and immunological signals [8]. The immune system is an important weapon for preventing tumorigenesis, progression, and metastasis and influences the tumor response to treatment. The immune system recognizes, controls, and kills cancer cells. However, cancer cells evade tumor immunity by decreasing autoimmunogenicity, constructing immunosuppressive networks, and suppressing immune responses [8]. The TME, which includes immunosuppressive networks, is associated with cancer progression, metastasis, and so on. Therefore, interventions that regulate autophagy in cancer cells and other cells in the TME by either promoting or inhibiting autophagy are considered effective antitumor therapeutic strategies and are widely used in the development of antitumor drugs.

Nanomaterials refer to materials with dimensions of 1 to 100 nm in at least one dimension of the 3-dimensional space, or materials composed of these dimensions as the basic unit. In recent years, due to their fine nanoscale size, excellent permeability and retention, low side effects, long circulation time, and broad targeting, nanomaterials have realized great potential in areas such as tumor therapy and tumor vaccine [8,9]. An increasing number of studies have shown that compared with traditional drug delivery methods, nanomaterial-constructed drug delivery systems can compensate for the shortcomings of traditional drugs, such as low stability and selectivity, improve the specificity for target cells, and improve therapeutic efficiency while reducing the toxicity of drugs and side effects [10]. In addition to delivering drugs, many nanomaterials have the ability to modulate cellular autophagy on their own due to the physicochemical properties of the materials themselves, such as photothermal effects. For example, several metallic nanomaterials (e.g., gold nanomaterials and zinc oxide) have been confirmed to cause cellular oxidative stress and activate cellular autophagic death through release. Some nanomaterials inhibit cellular autophagy by modulating the activity of kinases involved in the autophagy process and by disrupting lysosomal membrane permeability, thereby affecting autophagic flux [11].

**Fig. 1.**
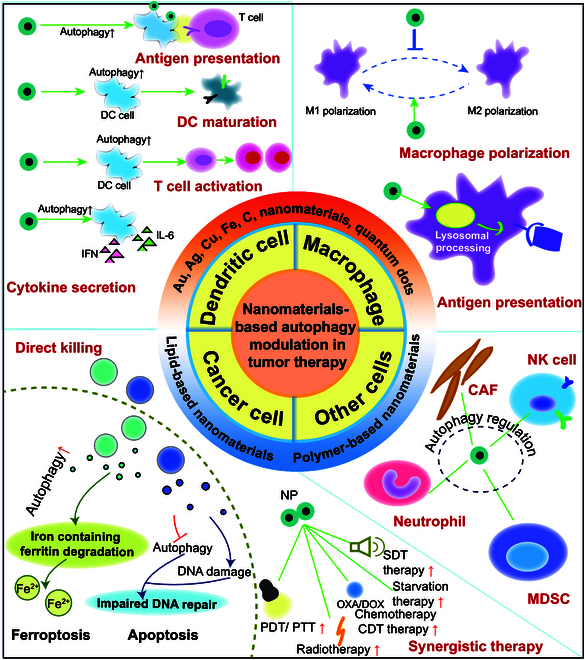
Nanomaterials modulate different cells through autophagy to promote antitumor resistance. To promote antigen cross-presentation, DC activation and maturation, T cell activation, macrophage polarization, and ultimately the direct or enhanced death of tumor cells via immunotherapy, nanomaterials control the autophagy of DCs, macrophages, tumor cells, fibroblasts, NK cells, and other cells. (Created with Adobe Illustrator.)

Leveraging the inherent properties of nanomaterials and their emerging capacity to modulate autophagy, the field of nanomaterial-based tumor therapy has undergone exponential growth. This review examines the burgeoning role of nanomaterials in the modulation of autophagy for anticancer applications. This study provides a comprehensive overview of the pivotal role that autophagy plays in tumor progression and the current landscape of autophagy-targeted cancer therapies. The classification of nanomaterials with autophagy-modulating potential and their distinctive characteristics are elucidated. Furthermore, this review delves into the intricate mechanisms by which these nanomaterials influence autophagy in tumor cells, tumor-associated macrophages (TAMs), dendritic cells (DCs), and other constituents of the TME (Fig. 1). The aim of this study was to provide a robust theoretical foundation and practical insights for the development of autophagy-controlled nanomaterials, recognizing the strategic importance of autophagy regulation in the advancement of cancer therapeutics.

## Autophagy and Cancer

Autophagy is a process of cellular self-destruction and is often regarded as a mechanism for cell survival. During tumor growth, autophagy selectively plays different roles. In the initial phases of malignancy, autophagy mainly inhibits the growth of tumor cells, whereas in the middle and late stages of tumor development, tumor cells in turn use the energy released by cellular autophagy to cope with hypoxia and malnutrition in the TME; at this time, autophagy plays a growth-promoting role in tumor cells [4]. Thus, modulating autophagy can effectively inhibit tumor cell growth and improve cancer treatment, which has been confirmed in several mouse models.

### The mechanism of autophagy

Autophagy is the act of transporting cytoplasmic cargo to lysosomes for degradation. Numerous signaling molecules and autophagy protein regulators, such as autophagy-associated genes (ATGs) and phosphatidylinositol 3-kinase (PI3K), participate in the autophagy pathway. These ATGs are regulated by intracellular mechanisms of nutrient acquisition, starvation, and stress, including mammalian target of rapamycin protein (mTOR), AMP activating protein (AMPK), and hypoxia-inducible factor (HIF) [12]. In the first phase, under starvation stimulation, ULK1 dissociates from mTOR1 and ATG12, AMPK is activated, and mTOR activity is reduced, eliminating the inhibitory effect on the ULK1 complex and initiating cellular autophagy, and phagolysosomes begin to nucleate [13,14]. In the second phase, during nucleation, the ULK1 complex activates the Beclin 1 (BECN1)-VPS34 complex, the VPS34 lipid kinase complex converts phosphatidylinositol (PI) to phosphatidylinositol 3,4,5-trisphosphate (PIP_3_), an isolation membrane is generated, and phagocytic vesicles are formed [15]. In the third phase, Atg7 activates Atgl2, causing it to temporarily bind first to Atg10 and then to Atg5, followed by heterodimerization and then binding to Atg6 to form the Atgl2–Atg5–Atgl6 complex. Moreover, Atg4 processes the precursor protein pro-LC3 into LC3-I, and LC3-I and PE pass through Atg7 and Atg3 to form LC3-II, which adsorbs to the membrane. In the fourth stage, the Atgl2–Atg5–Atgl6 complex detached from the outer membrane of the autophagosome, LC3-II was distributed on the bilayer membrane of the autophagosome, and the autophagosome slowly matured. In the fifth stage, autophagosomes and lysosomes generate autophagolysosomes, and the fusion of the 2 is accomplished under the synergistic effect of Rab, tethering, and the soluble NSF attachment protein receptor (SNARE) complex [16]. In step 6, the inner membrane of the autophagic lysosome and fusion proteins are broken down into small molecules, and the degraded material is released from autolysosomes and transported back to the cytoplasm via processes such as the recirculation of different cells [17].

### Autophagy in cancer

By eliminating damaged proteins and organelles, autophagy preserves cellular homeostasis and is crucial for the regulation of protein and organelle quality. Autophagy disruption has been linked to many types of human diseases, including cancer. In a range of circumstances and cancer development stages, autophagy can either stimulate or hinder the growth of tumors. Autophagy can prevent tumors from growing and can even prevent them from developing in the early stages of carcinogenesis. When tumors reach advanced stages, autophagy promotes tumor survival and growth, as well as the development of cancer metastasis. This section will summarize and discuss how autophagy suppresses tumorigenesis or promotes tumor development, metastasis, drug resistance, and stemness maintenance.

#### Tumor-suppressive functions

Normally, DNA damage or mutation will occur in response to the stimulation of carcinogens or the environment, which will induce the occurrence of tumors [18]. During this process, autophagy suppresses tumorigenesis by controlling protein and organelle quality, maintaining a stable intracellular environment, and inhibiting necrosis and inflammation. Several studies have demonstrated that deletion of autophagy promotes tumorigenesis [19–21]. Thus, early tumor suppression is significantly influenced by intact autophagy.

BECN1 is a key component of the PI3K complex, and the deletion of BECN1 gene has been found to be closely associated with the development of breast, prostate, and ovarian cancer [22,23]. Previous research has demonstrated that BCL2 binding to BECN1 can prevent autophagy and promote tumorigenesis, which confirms the inhibitory effect of BECN1 on tumorigenesis [24]. In addition, BECN1 can also bind to other molecules to regulate autophagy [25], such as UVRAG, which can promote autophagy and thus suppress tumorigenesis by binding to BECN137; Bif-1 promotes autophagosome formation by regulating BECN1 mainly through the interaction of its SH3 structural domain with UVRAG [26,27].

In addition, other autophagy-related proteins (ATGs), including ATG7, ATG9B, and ATG16L1, also play important roles in tumor suppression [18]. In non-small cell lung cancer, ATG7 can activate autophagy and thus inhibit tumor growth [28]. Similarly, the tumor-suppressive effects of ATG9B and ATG16L1 were found in cervical cancer and epithelial cell tumors, respectively. In summary, these findings imply that ATGs regulate autophagy, which in turn suppresses the growth of tumors [29].

SQSTM1/p62 is an autophagy-selective substrate and ubiquitin-binding protein. During the process of autophagy, SQSTM1/p62 can be specifically degraded after action, while defective autophagy leads to the accumulation of SQSTM1/p62, which damages mitochondria and DNA, increases oxidative stress, and further promotes tumor cell growth [30]. The development of cancer, particularly pancreatic, hepatocyte, lung, gastric, and breast cancers, is strongly linked to the accumulation of SQSTM1/p62 [31–33]. This finding suggested that the degradation of SQSTM1/p62 by autophagy may be one of the important mechanisms by which autophagy inhibits tumorigenesis at an early stage.

Inflammation initiates cancer development, and autophagy contributes to inflammation. Autophagy-deficient tumors show increased levels of inflammation, suggesting that autophagy defects may suppress inflammation, neoplasia, and cancer [18]. Autophagy disturbances increase the risk of normal tissue damage, necrosis, chronic inflammation, and genetic instability. These factors may also increase the risk of cancer development in the body.

#### Tumor-promoting functions

Early in the growth of a tumor, the organism does everything in its power to prevent tumor progression [34]. However, once a tumor reaches an advanced stage, autophagy can nourish the tumor cells, fostering treatment resistance, metastasis, and other features of tumor growth that are favorable for tumor survival.

Poillet-Perez et al*.* [35] indicated that the deletion of essential autophagy genes impairs tumor metabolism, proliferation, and survival and suggested that tumor suppression due to defective autophagy is associated with arginine deficiency. Furthermore, the role of autophagy in promoting tumor growth has been demonstrated in pancreatic, prostate, oral, and glioblastoma (GBM) cancers. These studies suggest that autophagy may support tumor cell survival and growth by providing nutrients. It inhibits inflammatory reactions, preserves genomic integrity, protects against DNA damage, and eventually stops cancer cell damage [36]. Dysfunctional mitochondria limit the ability of tumor cells to resist starvation, but autophagy is necessary to maintain healthy mitochondrial processes. Autophagy-deficient tumors exhibit morphologically irregular mitochondrial accumulation and dysfunction.

Autophagy is closely associated with tumor metastasis and is stage specific. By reducing inflammatory cell infiltration, autophagy may prevent tumor cell metastasis in the early tumorigenesis. However, in the later stages, autophagy may facilitate metastasis through adhesion, migration, invasion, and tumor cell detachment [18]. Zhao et al. [37] reported that autophagy can degrade paxillin and focal adhesion kinase in prostate, therefore encouraging tumor cell spread away from the original tumor location. In addition, epithelial–mesenchymal transition (EMT) enables epithelial cells to gain motility and metastatic potential and is key in tumor metastasis [38]. On the one hand, autophagy can provide energy and essential nutrients for EMT during metastatic. On the other hand, autophagy may mediate cytoskeletal remodeling by regulating signaling pathways such as WNT, NF-kB, and TGF-β64, which may contribute to EMT progression [38].

Increased autophagy may cause medication resistance in a variety of tumor types in addition to promoting the survival, growth, and spread of tumor cells [39,40]. For instance, high expression of WASF3 enhances autophagy, which in turn causes oxaliplatin resistance in gastric cancer cells [41]. In addition, autophagy can reduce metabolic, oxidative, and endoplasmic reticulum (ER) stress, thus promoting the development of drug resistance [42]. The mechanism of antitumor drugs mainly involves the induction of tumor cell apoptosis, while autophagy-mediated drug resistance may involve drug concentration, gene repair, apoptosis, and survival signals [39]. In conclusion, autophagy inhibition seems to be an important approach for preventing tumor drug resistance at present. However, recently, more studies have indicated that autophagy inhibition does not attenuate drug resistance in certain tumors [43,44]; therefore, the effect of autophagy on tumor drug resistance may be related to different types and stages of tumors, and further studies are needed.

Furthermore, autophagy can aid in blocking the immune system from identifying tumor cells by decreasing host antigen presentation, destroying major histocompatibility complex class I (MHC-I), and inhibiting T cell identification [7]. Yamamoto et al*.* [45] reported that the MHC-I levels in pancreatic ductal adenocarcinoma cells were lower than those in normal pancreatic cells, while their lysosomes showed a high enrichment of MHC-I molecules. Autophagy inhibition in pancreatic ductal adenocarcinoma cells using autophagy inhibitors resulted in a significant increase in MHC-I levels and CD8^+^ T cells [45]. Therefore, restoring MHC-I levels on the surface of cancer cells and boosting anticancer T cell responses in vivo can be accomplished through autophagy inhibition [7].

In addition to mediating MHC-I degradation, autophagy also influences immune cell migration and chemokine production in tumor cells [7,46]. A study in 2019 showed that pancreatic cancer cells deficient in the autophagy gene Atg5 produced higher levels of chemokines and attracted more immune cells to pancreatic tumor tissue in mice than did pancreatic cancer cells with intact autophagy [47]. Moreover, autophagy has been demonstrated to guarantee the life of tumor cells by stopping immune responses and decreasing the ability of natural killer (NK) cells and CD8^+^ cells to eliminate tumors [48]. In conclusion, autophagy can not only provide nutrients to tumor cells and prevent cell damage but also help tumor cells evade the host immune response to maintain their long-term survival and growth and promote tumor growth by destroying MHC and inhibiting chemokine expression.

Autophagy plays a dual role in the developmental stage of tumors. In the early stage of tumors, autophagy acts as an assistant of the body’s immunity to inhibit tumor growth and proliferation; however, when the tumor breaks through the limitations of the body’s immunity and continues to grow and proliferate to the advanced stage of tumors, autophagy turns into an assistant of the tumor to provide energy and nutrients to the tumor, at which time the inactivation of autophagy is essential to minimize the damage to the tumor and the metastasis is crucial. In addition, autophagy has been associated with the maintenance of tumor stem cell properties and drug resistance in tumor cells. Autophagy is emerging as a viable target for cancer therapy, and finding accurate ways to precisely regulate autophagy is a promising strategy for cancer treatment.

Nanomaterials represent a promising therapeutic modality for tumor intervention, exerting their influence by modulating the autophagic process. Research has demonstrated that nanoparticles (NPs) regulate autophagy-related proteins in certain ways, such as LC and BECN1. Both soft nanomaterials, represented by liposomes, and hard nanomaterials, represented by metals, can use their respective material qualities to regulate autophagy-related proteins and increase oxidative stress, which will slow the growth of cancer. Furthermore, pharmaceuticals can be transported using nanomaterials as carriers. Additionally, the combination of autophagy-regulating medications with nanomaterials can enhance drug delivery targeting, boost drug accumulation in tumor cells, and enhance drug usage. This comprehensive review delineates 5 pivotal classes of nanomaterials, elucidating their mechanisms for modulating autophagy to enhance therapeutic efficacy in oncological interventions.

## Nanomaterials for Autophagy Regulation and Tumor Therapy

Most nanomaterials used for diagnostic and therapeutic purposes in the field of biomedicine have a particle size of 10 to 100 nm, and have different magnetic, electrical, and photothermal properties than traditional materials [49]. These advantages cause the nanomaterials themselves to have certain effects on cells, and the use of material properties can block cell signaling pathways, and induce immune cells, thus achieving the purpose of tumor therapy. For example, photothermal properties can be used to increase the temperature of the environment around the cell to kill tumor cells while inhibiting the proliferation and differentiation of tumor cells. Furthermore, through material degradation, DC cells are recruited in situ to trigger targeted antitumor immunity and prevent tumor recurrence [50].

It has been found that nanomaterials, mainly represented by metal NPs, can utilize their own material properties to induce oxidative stress-dependent signals and can also accumulate in the cells, thus activating lysosomes, inducing cellular autophagy and cancer treatment [48, 51, 52]. In addition, some nanomaterials can disrupt cellular autophagy by blocking cellular autophagic flow, disrupting cellular autophagy function, disrupting cellular clearance of foreign substances, and leading to the death of tumor cells, thus disrupting cellular autophagy. This statement has been confirmed and supplemented by previous studies in our group [53].

As common drug carriers, nanocarriers are usually synthesized in the form of single or multiple materials combined to transport tumor therapeutic drugs. Compared with traditional drug delivery systems, nanomaterial-based platforms have the advantages of controlled release, high bioavailability, strong targeting activity, and versatility [49]. In our previous research, we have developed a variety of drug delivery systems that achieve controlled release of drugs and at the same time make the materials biodegradable, improve the precision and therapeutic efficacy of the drugs, and reduce the side effects of the treatment on the organism [54]. Endogenous stimuli [e.g., pH response and reactive oxygen species (ROS) response] or exogenous stimuli (e.g., light response, heat response, and ultrasonic response) can induce stimuli-responsive drug release, thereby reducing toxic side effects [51]. However, these endogenous or exogenous stimuli are not exclusively tumor specific, so more precise stimuli need to be developed to achieve true on-demand release. The DNA cross-linked mucin nanomaterial nanocarriers constructed by Kimna et al*.* [52] are capable of triggering release from tumor cells. Through the displacement of specific nucleotide sequences, the release of drugs within nanocarriers can be triggered to recognize target cells at the molecular level [52]. These findings highlight the outstanding advantages of nanocarriers for precision drug release.

Targeting is another major advantage of NP material-based drug delivery systems compared to free drugs. Targeted drug delivery strategies aim to precisely deliver drugs to specific tumor cells or tissues through passive targeting or active targeting. However, these 2 targeting strategies differ in terms of uptake rates, and MacCuaig et al*.* [55] showed that in situ pancreatic cancer cells had a greater uptake rate of V7 peptide-mediated active-targeting NPs. In conclusion, active targeting further enhances the hyperosmotic long retention effect and intracellular internalization process of intratumor drug delivery, which is a major advantage of NPs for tumor treatment [49].

In this section, we will introduce bio-nanomaterials relevant to tumor therapy and the mechanisms by which they exert their therapeutic effects on tumors, according to a classification criterion based on material structure (Table 1).

**Table 1.** Nanomaterial-modulated autophagy in cancer therapy

Nanoparticles	Autophagy marker (up/down)	Mechanisms	Ref.
Polymeric nanoparticles	JNK↑, Akt/mTOR↓	Increasing ROS levels, activating JNK signaling, and suppressing Akt/mTOR signaling Triggering apoptosis and autophagy in cancer cells	[56]
Polymeric nanoparticles	LC3-II↑ P62↑	Autophagy inhibition using chloroquine promotes cell death	[57]
Polymeric nanoparticles	Beclin 1↑	Triggering autophagy to potentiate cell death in cancer cells	[58]
Polymeric nanoparticles (PLGA and succinate)		Inhibiting autophagy to protect doxorubicin and enhancing nuclear translocation of this chemotherapeutic agent	[59]
Polystyrene nanoparticles		Autophagy inhibition promotes antitumor activity	[60]
Polymeric nanoparticles	LC3-II↑	Enhancing accumulation of autophagosomes in triggering autophagy	[61]
Polymeric nanoparticles		Decreasing sorafenib-mediated autophagy by delivery of HGFK1	[62]
Polymeric nanoparticles		Inducing both apoptosis and autophagy in reducing the viability of cancer cells	[63]
Lipid nanoparticles	Beclin 2↓	Down-regulation of Bcl-2 expression in endoplasmic stress to trigger autophagy	[64]
Titanium dioxide H	LC3II, p62, NBR1, Beclin 1, and ATG5	Autophagy induction at low dose; autophagy blockage at high dose	[65]
Gold nanoparticles	LC3↑, p62↑	Autophagy blockade and lysosomal dysfunction	[66]
Au nanoparticles	LC3-II↑	Down-regulation of mTOR and PI3K/Akt, up-regulation of LC3-II and ERK, inducing autophagy	[67]
AgNPs		Induction of autophagy, autophagosome accumulation	[68]
Silver nanoparticles	LC3↑, p62↑	Autophagy blockade and lysosomal dysfunction	[69]
Silver nanoparticles	PtdIns3K	Autophagy induction; inhibition of autophagy enhances the anticancer activity of silver nanoparticles; tumor killing mediated by photothermal effect	[70]
Au–Ag nanoparticles	Akt, ERK	Providing photothermal therapy and enhancing ROS levels, triggering Akt and ERK signaling pathway, stimulating both autophagy and apoptosis	[71]
Fe3O4 NPs	LC3↑	In vitro: mitochondrial dysfunction, excessive ROS, DNA damage, cell death; in vivo: tumor growth inhibition	[72]
Fe3O4 NPs	LC3↑	In vivo: inhibition of tumor growth, ROS, mitochondrial damage necrosis, autophagic cell death	[73]
Iron oxide	Akt-AMPK-mTOR	Selectively induce autophagy and kill cancer cells	[74]
Copper oxide	LC3↑, p62↑	Autophagy blockade and lysosomal dysfunction	[75]
CuS NPs	LC3↑	Induction of autophagy	[76]
CuS NPs	LC3↑	Autophagy blockage	[77]
ZnO NPs	LC3↑	Autophagy induction, autophagosome accumulation	[78] [79]
ZnO NPs	LC3↑, Beclin 1↑, P62↓	ROS, mitochondrial dysfunction, cell death	[80]
Fullerene C60	Atg5	Autophagy-mediated chemosensitization in cancer cells	[81]
Graphene oxide	PtdIns3K and MEK/ERK1/2	Activation of autophagy and elimination of ubiquitinated mutant Huntington protein	[82]
Graphene oxide	LC3↑, p62↑	Autophagy blockade and lysosomal dysfunction	[83]
Graphene oxide QDs	LC3↑, p62↑	Autophagy blockade and lysosomal dysfunction	[84]
CdTe and CdTe/CdS/ZnS QDs	LC3↑	Autophagy sensitized cytotoxicity	[85]
Single-walled carbon nanotubes	mTOR-S6K	Autophagy induction and lysosomal activation	[86]

### Metal nanomaterials

According to their chemical composition, nanomaterials can be categorized into nanometallic materials, nano-organic materials, and nanocomposites. Among them, nanometallic materials refer to metal and alloy materials that form nanocrystalline grains, which are usually characterized by grain boundary ratios, specific surface energies, and large ratios of surface atoms. Common nanometallic materials and their oxide materials include gold nanomaterials, silver nanomaterials, copper oxide, iron oxide, and zinc oxide. These materials usually have basic properties such as electrical conductivity, superplastic ductility, catalysis, and antimicrobial properties. According to the study, in terms of antimicrobial properties, silver, copper, zinc, and other nanometals and their oxides have been shown to induce oxidative stress, with spectroscopic bactericidal effects. In addition, a number of nanomaterials, such as gold, silver, copper, iron, zinc, and their oxide materials, have been demonstrated to modulate cellular autophagy.

Colloidal gold, another name for gold NPs (AuNPs), is a nanomaterial with exceptional biocompatibility. In biomedicine, gold has long been considered an inert precious metal with unique properties, such as good biocompatibility and few side effects, and thus has high therapeutic value. Because of their unique material properties, AuNPs are widely used in drug delivery [56], imaging diagnostics [57], tumor photothermal therapy [58], and radiotherapy sensitization [59]. In addition, AuNPs are regulators of cell fate and participate in cell death-related regulation; thus, AuNPs play a supplementary role in inducing apoptosis and autophagy and synergistically treating tumors when used as carriers or exogenous stimulants to treat diseases [60–61].

Gold nanomaterials themselves possess the ability to control cellular autophagy, and this effect can be used to induce autophagy-related cell death on the one hand as a direct antitumor effect, and on the other hand, they can be used to sensitize cells to radiotherapy as an adjuvant tumor therapy. AuNPs can block the autophagic flow of cells and impair the autophagic function of cells because of the lysosomal alkalinization of AuNPs after they are phagocytosed, which leads to the blockage of lysosomal degradation ability and fusion with autophagic vesicles to form autophagic lysosomes, known as impaired lysosomes [63].

**Fig. 2.**
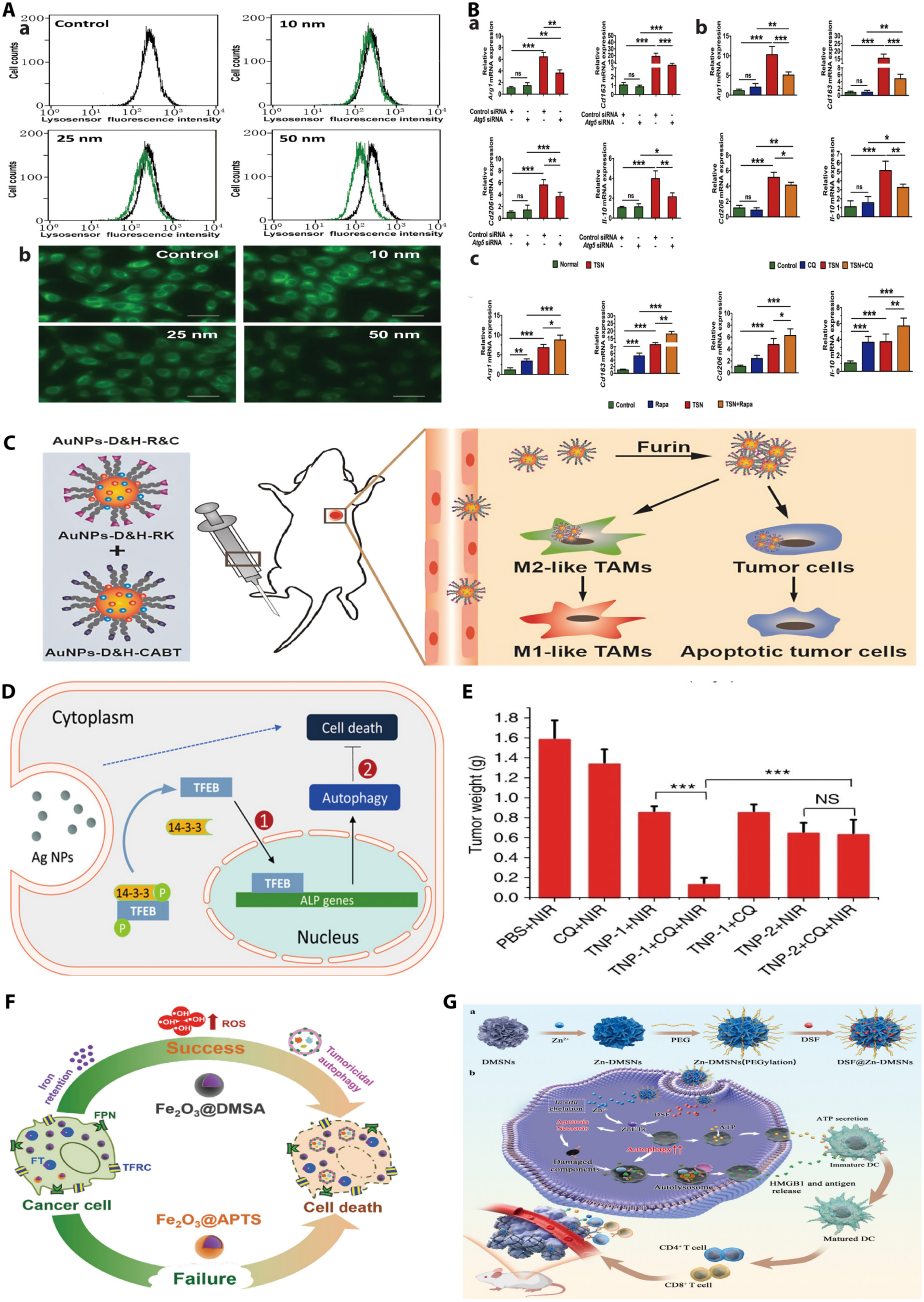
Metallic nanomaterials for the treatment of tumors by influencing autophagy. (A) Effect of AuNPs on lysosomal pH. (a) Fluorescence-activated cell sorting (FACS) analysis of stained cells. Black line, control cells; green line, experimental cells. (b) Representative fluorescence images of normal rat kidney (NRK) cells treated with AuNPs for 24 h and then exposed to the dye for 30 min [64]. (B) Inhibition of M2 macrophage polarization by autophagy blockade. Analysis of relative mRNA expression of Arg1, Cd163, Cd206, and Il-10 in TSN co-cultured RAW 264.7 cells incubated with Atg5 siRNA (a), chloroquine (CQ) (b), and rapamycin (Rapa) (c) [65]. (C) Diagram of the mechanism by which AuNPs-D&H-R&C are administered intravenously to mice [71]. (D) Mechanism underlying AgNP-induced TFEB translocation and cytoprotective autophagy [77]. (E) Autophagy inhibitors significantly enhanced the anticancer effects of TNP-1-mediated PTT in the 4T1 model. Tumor volume of mice in each group on day 15 of treatment [96]. (F) Carboxyl-functionalized iron oxide NPs (Fe_2_O_3_@DMSA) significantly affected iron transport systems [transferrin receptor (TFRC), ferritin (FT), and ferroportin (FPN)], promoting intracellular iron retention and excessive ROS-induced tumor autophagy [87]. (G) Schematic of the ability of the DSF@Zn-DMSN construct to improve cancer chemoimmunotherapy efficacy [98]. Reproduced with permission [64]. Copyright 2011, American Chemical Society. Reproduced with permission [65]. Copyright 2022, Elsevier Ltd. Reproduced with permission [71]. Copyright 2021, Elsevier Ltd. Reproduced with permission [77]. Copyright 2020, Wiley. Reproduced with permission [97]. Copyright 2018, Wiley. Reproduced with permission [87]. Copyright 2018, PubMed Central. Reproduced with permission [98]. Copyright 2023, Elsevier Ltd.

The blockage of autophagic flux increases the accumulation of autophagic vesicles in the cell, which disrupts the removal of foreign substances and ultimately leads to cell death. Ma et al*.* [64] created AuNPs by synthesizing them with chloroauric acid and sodium citrate. They conducted experiments using mouse kidney cells and found that AuNPs led to the accumulation of autophagosomes by affecting cellular endocytosis and lysosomal function. This study revealed that AuNPs hinder lysosomal degradation by interfering with autophagosome–lysosome binding, causing autophagosome buildup and lysosomal enlargement in cells. Research has shown that the uptake of AuNPs by cells and the induction of autophagosome accumulation are influenced by the size of the particles; larger NPs are taken up more quickly and induce a stronger response, potentially blocking the fusion of autophagosomes with lysosomes and reducing lysosomal degradation. The study also revealed that treatment with AuNPs did not directly induce autophagy but rather impacted autophagy by disrupting autophagic flux, leading to autophagosome accumulation. Further investigations confirmed that the accumulation of AuNPs in lysosomes led to the alkalinization of lysosomes, disrupting their degradation function and causing the expansion of lysosomes and the blockage of autophagic flux in cells [64]. In addition to affecting cellular lysosomes in macrophages, NPs can influence T cell activation and antitumor functions by affecting processes such as antigen presentation in DCs (Fig. 2A).

Our research group utilized AuNP materials as a foundation to explore the use of polyethylene glycol nanomaterials for modification (PEG-AuNPs) to enhance their impact on autophagy in TAMs and inhibit the M2-type polarization of TAMs, ultimately aiming to achieve antitumor immunotherapy [65]. Specifically, these materials cause macrophage lysosome alkalinization and changes in cell membrane permeability upon accumulation, resulting in the obstruction of autophagic pathways, a reduction in autophagic flux, and the suppression of autophagy in TAMs. The matching in vitro and in vivo experiments demonstrated a noteworthy reduction in the recycling rate of the autophagy-related protein LC3. To investigate the impact of autophagy on TAM polarization, tumor culture supernatant (TSN) macrophages were cocultured with autophagy inducers (ranibizumab) and inhibitors [chloroquine (CQ) and Atg5 small interfering RNA (siRNA)], and autophagy inhibition led to a decrease in M2-type polarization in TSN-cocultured macrophages, while autophagy induction promoted M2-type polarization. This finding elucidates how PEG-AuNPs inhibit M2-type polarization in TAMs and suggests an alternative approach to leveraging macrophage autophagy for antitumor effects [65]. Furthermore, our investigation of the effects of these materials on DCs and T cells revealed that PEG-AuNPs stimulated DC maturation, increased the CD4^+^ and CD8^+^ T cell ratio, and activated and enhanced antitumor responses within the body (Fig. 2B).

These studies have investigated the effects of AuNPs of different sizes on autophagy for synergistic therapeutic effects. Metal NPs, such as cadmium and silver, are known to have strong cytotoxic and oxidative stress effects on cells. However, the potential biotoxicity of AuNPs remains unclear. In a study conducted by Li et al*.* [66], MRC-5 human fetal lung fibroblasts were utilized to examine the effects of AuNPs. The findings revealed that AuNPs triggered cellular autophagy and oxidative stress in the cells. Specifically, the uptake of AuNPs by the cells in the experimental group led to the formation of autophagosomes and an increase in the expression of the autophagy gene ATG7 and the autophagy-related protein LC3-1, indicating enhanced cellular autophagy. Moreover, the up-regulation of PNK suggested that the damaged DNA repair mechanism remained functional in the cells. Elevated levels of malondialdehyde (MDA) and the up-regulation of genes such as PRP and OSRI further demonstrated that the cells activated an antioxidative stress protection mechanism [66]. The combined results of the experiments suggest that AuNPs induce cellular autophagy and oxidative stress while activating protective mechanisms against oxidative stress. It is postulated that autophagy may serve as a cellular defense mechanism against oxidative stress toxicity.

In addition to damaging lysosomes, AuNPs can also damage mitochondrial membranes to release ROS, causing an oxidative environment, and autophagy protects cells from oxidative stress; therefore, the application of AuNPs is often accompanied by oxidative stress and autophagy [66]. In 2011, Wu et al*.* [67] used perovskite-cored gold-shelled NPs to explore the mechanism of mitochondria-mediated autophagy in the selective killing of tumor cells by NPs. This selectivity was mainly attributed to the activation of autophagy due to the impaired mitochondrial membrane potential of tumor cells. They also found that NPs at concentrations between the half-maximal inhibitory concentration (IC_50_) and inhibitory concentrations causing 20% (IC_20_) kill tumors only by inducing autophagy, while cytotoxicity at concentrations below the IC_50_ may be related to ROS production. In a study by Dhandapani et al*.* [68], AuNPs biosynthesized from Nigella sativa and Curtobacteriumproimmune K3 were considered to have potential for development as antitumor drugs because of their ability to generate ROS, disrupt mitochondrial membranes, and act as a blocker of autophagic flow in human gastric adenocarcinoma cells (AGS), inducing cell death. The different responses of tumor cells and normal cells to oxidative stress lead to differences in the activation of autophagy, which is an important mechanism by which NPs selectively kill tumor cells to reduce damage to normal cells.

In the field of tumor therapy, this blockade of autophagy could be used to improve tumor efficacy. X-ray irradiation-induced protective autophagy and the ability of tumor cells to repair damaged DNA are key factors contributing to the ineffectiveness of radiotherapy in GBM patients. Therefore, overcoming cellular radioresistance is crucial for enhancing the efficacy of radiotherapy. Au@Cu_2−*x*_Se NPs, or core-shelled copper selenide-coated AuNPs, were the subject of a study by Xu et al*.* [69]. Researchers discovered that Au@Cu_2−*x*_Se NPs could modulate lysosomal intracellular pH, induce lysosomal alkalinization, impede autophagic flux, and suppress protective autophagy in tumor cells by utilizing AuNPs to obstruct autophagy and harm lysosomes with hydrogen peroxide. Additionally, it was shown to stimulate the ubiquitination of Rad51 (a DNA repair protein), enhance the proteasome-mediated degradation of Rad51, and inhibit DNA repair in tumor cells. It increased SQSTM1/p62 protein levels in tumor cells without changing cellular mRNA levels. These findings indicated that the Au@Cu_2−*x*_Se NPs enhanced tumor therapy by preventing autophagy and DNA repair, increasing the susceptibility of tumor cells to radiation, diminishing cellular repair mechanisms, and enhancing the effectiveness of radiotherapy. Apart from the inherent autophagy-modulating properties of AuNPs, surface-loaded autophagy-modulating drugs can also regulate cellular autophagy levels [69]. For instance, Ma et al*.* [70] utilized surface-modified AuNPs with a diameter of approximately 100 nm as a sensitizer for radiotherapy in an animal model of cervical cancer. The results demonstrated that AuNPs induced cellular autophagy, resulting in radiotherapy resistance, whereas the presence of the loaded autophagy inhibitor 3-methyladenine (3-MA) could enhance tumor responsiveness to radiotherapy and increase the efficacy of tumor treatment.

In addition to being used as direct autophagy modulators, AuNPs are able to synthesize nanocarriers and construct drug delivery platforms to indirectly regulate cellular autophagy by delivering autophagy-modulating drugs and improving drug utilization. Xie et al*.* [71] designed a furan-responsive aggregation nanoplatform (AuNPs-D&H-R&C) loaded with doxorubicin (DOX) and hydroxychloroquine (HCQ), which is capable of simultaneous chemotherapy, autophagy regulation, and immune microenvironment remodeling. The acidic environment of tumors and lysosomes facilitates CQ release, inhibits cellular autophagy to restore tumor cell sensitivity to chemotherapeutic agents, and alters M2-type TAMs to M1-type macrophages, which suppress tumors, to work in concert to exert anticancer effects (Fig. 2C) [71].

The effect of AuNPs on autophagy is a complex process related to the particle size, shape, and concentration of AuNPs [64,72,73]. AuNPs can synergistically exert antitumor effects by modulating autophagy to sensitize other tumor therapeutic strategies, modulating autophagy levels in the immune microenvironment, and disrupting mitochondria to activate autophagy. If properly utilized, the modulation of autophagy by AuNPs could play a beneficial role in tumor therapy. The modulation of autophagy by AuNPs and their role in the sensitization to radiotherapy make AuNPs a promising therapeutic agent for tumor therapy, so the influence of AuNPs on autophagy should be fully considered when designing AuNPs as therapeutic agents for tumor therapy.

Silver NPs (AgNPs) are cytotoxic to cancer cells and possess excellent potential as an antitumor agent [74]. The role of AgNPs in regulating autophagy is also of increasing interest to researchers. Wen and Gu’s research team [75] has made many contributions to the regulation of autophagy induced by AgNPs, and they suggest that AgNPs, in addition to their cytotoxic effects, could induce protective autophagy in tumor cells and antagonize the therapeutic effects of drugs or radiotherapy. It has been reported that AgNPs can induce cellular autophagy by activating the PtdIns3K signaling pathway, and this autophagy does not destroy lysosomes to affect foreign body degradation to block autophagic flow but promotes increased autophagosome formation. The autophagy induced by AgNPs is cytoprotective autophagy, a prosurvival mechanism for tumor cell self-protection, so the combined use of ATG5 gene knockdown and the autophagy inhibitor wortmannin can improve the therapeutic benefit of AgNPs [75,76].

Research studies have shown that nanosilver can lead to many cell biological effects, such as oxidative stress, DNA damage, apoptosis, and necrosis. AgNPs are cytotoxic to cancer cells and possess excellent potential as an antitumor agent. The research team of Wen and Gu [75] made many contributions to the induction of autophagy by AgNPs in antitumor regulation, and they concluded that AgNPs could induce protective autophagy in tumor cells and antagonize the therapeutic effects of drugs or radiotherapy, in addition to their cytotoxic effects. It has been reported that AgNPs can induce cellular autophagy through activation of the PtdIns3K signaling pathway, which does not destroy lysosomes to affect foreign body degradation to block autophagic flow, but rather promotes increased autophagosome formation. AgNP-induced autophagy is cytoprotective autophagy, a pro-survival mechanism for tumor cell self-protection, and therefore, the combination of ATG5 knockdown and the autophagy inhibitor wortmannin can improve the therapeutic efficacy of AgNPs [75,76].

To further investigate the mechanism by which AgNPs induce cytoprotective autophagy, Wen’s team [77] found that dephosphorylation of TFEB at serine-142 and serine-211 promoted its increased translocation into the nucleus, leading to increased expression of autophagy-related genes, and elucidated the regulatory mechanism by which AgNPs promote autophagy (Fig. 2D). Similarly, AgNP-induced protective autophagy has been reported to occur during the sensitization of gliomas to radiotherapy. While promoting protective autophagy, AgNPs were able to increase the sensitivity of cells to radiation therapy. When 3-MA was used to suppress this protective autophagy, the rate of apoptosis in U251 cells increased. The authors also further demonstrated that the activation of protective autophagy was related to the extracellular signal–regulated kinase (ERK) and c-Jun N-terminal kinase (JNK) pathways [78]. The team further investigated the mechanism of AgNP-induced radiosensitization and found that ROS production is a key factor in radiosensitization, and the inhibition of protective autophagy by 3-MA is also believed to increase ROS levels, ultimately leading to increased sensitivity of glioma cells (GCs) to radiotherapy [78]. These studies have deepened our understanding of the mechanism of AgNP-induced pro-survival autophagy and provided a theoretical basis for the use of AgNPs combined with autophagy inhibitors for anticancer therapy.

On the other hand, silver ions released from AgNPs can cause cellular mitochondrial damage and increase ROS release to initiate autophagy [76,79]. Ghaemi et al. [80] reported that ultraviolet (UV) radiation-induced Ag@ZnO NPs can cause ROS damage to the Golgi, which induces excessive cellular autophagy and ultimately leads to melanocyte apoptosis. This ROS release is generally considered to be associated with mitochondrial damage caused by AgNPs [81]. A study conducted by Manshian et al*.* [82] revealed that multiple tumor cell lines (e.g., KLN205, A549, and HeLa) exhibit AgNP concentration-dependent oxidative stress, mitochondrial damage, and autophagy induction. In addition to AgNPs, polymer-coated hybrid AgNPs can also increase autophagy in tumor cells. Polymeric metal NP complexes such as gold–silver nanoparticles encapsulated with polydopamine are capable of mediating tumor killing through photothermal effects and are also capable of resulting in both the activation of autophagy and mitochondrial damage in tumor cells [83].

The cytotoxicity of nanosilver is a subject of ongoing scientific inquiry, with 2 predominant mechanisms currently under discussion. One perspective posits that nanosilver particles dissolve in cell culture media, releasing Ag^+^ ions that exert a toxic effect on cells. The alternative viewpoint suggests that these particles can internalize into cells through fusion with cellular organelles or phagocytosis, interacting with these organelles and causing disruption of the cytoskeleton, interference with normal cellular functions, and potentially leading to cell necrosis [84,85]. Upon entering the human body, AgNPs are hypothesized to circulate through the bloodstream or lymphatic system, reaching secondary target organs and inducing cytotoxic effects beyond the primary target cells. Therefore, a key focus of current research is to mitigate the toxic effects of nanosilver on normal body cells while leveraging its tumor-killing potential [76]. It has been observed that nanosilver with different particle sizes and at various dose/concentration ranges elicit different effects on the organs of living organisms. The development of nanosilver with smaller particle sizes may be an effective strategy to address cytotoxicity. Moreover, studies, including those by Wen et al. [75], have demonstrated that the co-administration of nanosilver with autophagy inhibitors can significantly enhance the antitumor efficacy of nanosilver at the same concentration. This suggests that the combined use of autophagy modulators could reduce the dose/concentration range of nanosilver required, accelerate its metabolism within the body, and decrease the cytotoxic effects associated with its accumulation. Lastly, it has been proven that the cytotoxicity varies depending on the route of administration, and improving the targeting of nanosilver to tumor tissues can also effectively reduce the dose/concentration range utilized.

Elevated levels of cellular autophagy induced by Fe_2_O_3_ NPs are very common in tumor cells, and Fe_2_O_3_ NPs are considered promising therapeutic nanomaterials for a wide range of applications, such as drug delivery, anemia, and magnetic resonance [60,86]. Unlike gold and AgNPs, which induce tumor-killing autophagy by codelivering autophagy modulators (e.g., CQ and HCQ), Fe_2_O_3_ NPs are believed to induce tumor-killing autophagy by stimulating cells to release large amounts of ROS and disrupting cellular autophagic flow. Considering the toxicity of autophagy inhibitors and their nonselectivity for cells, Xie et al*.* [87] designed 2 types of autophagy inhibitors [87]. The results showed that the intracellular aggregation of Fe_2_O_3_@DMSA (a carboxy‐functional iron oxide nanoparticle; DMSA, dimercaptosuccinic acid) induced sustained ROS production in cells, which led to the fusion of autophagic vesicles with lysosomes and triggered tumor-killing autophagy (Fig. 2F). Moreover, unlike autophagy inhibitors, this type of death-promoting autophagy induction is thought to be tumor cell-selective; for example, in the lung, iron oxide NPs synthesized by Khan et al*.* [88] selectively induced autophagy in human lung cancer A549 cells without significant toxicity to normal human lung fibroblasts (IMR-90). This selectivity is one of the main advantages of iron oxide NPs in treating tumors by modulating autophagy.

In addition to their application in tumor therapy, iron oxide NPs have a wide range of applications in tumor diagnostics, especially in magnetic resonance imaging (MRI). For example, superparamagnetic iron oxide NPs (SPIONs), biocompatible and nontoxic iron NPs, are U.S. Food and Drug Administration (FDA)-approved MRI contrast agents [89]. Zhang et al*.* [90] designed a Fe_3_O_4_ NO· NPs (NO· radical-conjugated Fe_3_O_4_ nanoparticles; NO·, nitric oxide free radical) that facilitates enhanced T1- and T2-weighted imaging in stable intracellular environments, where autophagy occurs by stimulating cells to produce a large amount of reactive substances, which reduces the T1 signal of NO-203. Thus, the signal of autophagic flow can be reflected by the ratio of T1 signal intensity to T2 signal intensity, and this study provides a viable method for visualizing autophagy in vivo.

Although several SPIONs have received clinical approval for medical applications, the potential toxicity associated with these particles remains a contentious issue [91,92]. Empirical evidence has delineated a spectrum of cytotoxic mechanisms associated with SPIONs, including the generation of ROS, disruption of mitochondrial function, induction of apoptotic vesicle formation, and DNA damage [93]. Notably, oxidative stress precipitated by SPION-induced ROS accumulation within the cellular milieu has emerged as a pivotal factor in cytotoxicity. The intrinsic composition, particle size, concentration, and surface functionalization of SPIONs are paramount in determining their toxicological profile. Specifically, the size of SPIONs is posited to be a critical determinant for systemic administration, with a consensus favoring a size range of 10 to 100 nm as optimal [93]. Furthermore, the cytotoxic potential of SPIONs is significantly influenced by the nature of their surface coatings. The size, surface charge, and coating are pivotal in modulating the absorption and distribution dynamics of SPIONs. In the wake of burgeoning advancements in surface modification technologies, researchers are tirelessly endeavoring to explore drug delivery strategies that could substantially enhance the safety profile of these materials.

Many studies have reported the use of Cu NPs for tumor therapy. Additionally, in recent years, scientists have gained a better grasp of how copper functions in cancer treatment. Traditionally, Cu is considered a static cofactor within the active site of the enzyme; however, according to current research, Cu is necessary for the autophagic kinases ULK1 and ULK2 (ULK1/2) to function, and in the case of KRASG12D-driven lung cancer, Cu can enhance autophagic flux to promote tumor growth and survival [94]. However, the effect of exogenous Cu NPs on the level of cellular autophagy after application is mainly reflected in the macroautophagy response of cells to Cu NPs rather than the aforementioned pathway regulation, and the regulatory effect of Cu NPs on autophagy has been the subject of very few investigations. In this section, we will examine the literature on this topic.

Cu NPs can exert synergistic antitumor effects when combined with chemotherapeutic agents. Xiong et al*.* [96] revealed the potential mechanism by which copper NPs can induce cytotoxicity via the induction of apoptosis and autophagy, which have synergistic antitumor effects when combined with cisplatin and gemcitabine. Although chemotherapy remains one of the main strategies for oncological treatment, the use of these chemotherapeutic agents still leads to multidrug resistance in tumors, and the use of nonchemotherapeutic agents in combination with physiotherapy (e.g., photothermal therapy) offers a new way to solve this dilemma. For instance, Zhang et al. [96] developed a Cu–Pd alloy via a combination of chemotherapeutic and photothermal therapy (PPT) approaches. In contrast to traditional chemotherapy, autophagy inhibitors such as CQ or 3-MA have been used to suppress pro-survival autophagy and improve the efficacy of photothermal therapy. They also found that autophagy is dependent on shape and composition and that the structure of the sharp-tip structure within this material and Cu were indispensable for the induction of autophagy (Fig. 2E) [96]. In addition, there have also been improvements in drug delivery strategies to overcome the multidrug resistance of tumors. For example, Xiong et al*.* [97] prepared Aft-Cu using apoferritin, an endogenous natural nanocarrier encapsulated with Cu, and the nanomaterial demonstrated strong anticancer efficacy against multidrug-resistant colon cancer cells and was able to cause cell death by triggering autophagy-dependent apoptosis.

Our research group has also directed attention toward leveraging metallic zinc for the enhancement of antitumor immune responses, in addition to the commonly studied metal nanomaterials [98]. In particular, we created dimethyl disulfide (DSF)-loaded zinc-doped dendritic mesoporous silica NPs (Zn-DMSNs). The slightly acidic TME receives Zn^2+^ and DSF upon pH-triggered breakdown of DSF@Zn-DMSNs, which produces poisonous Zn^2+^ and promotes pH-responsive zinc ion release. Furthermore, zinc ions can form ZnETs via ion chelation reactions, which not only exhibit strong therapeutic effects but also trigger the formation of chemicals known as damage-associated molecular patterns (DAMPs), such as ATP, CRT, and HMGB1, as well as autophagy in tumor cells. These DAMPs can stimulate immunogenic cell death (ICD), initiate immune cell death signals, advance DC maturation, and improve T cell infiltration, thereby bolstering the efficacy of tumor chemoimmunotherapy while minimizing adverse effects on nontarget organs (Fig. 2G) [98]. Our study highlights autophagy as a pivotal pathway for immunogenic cell death activation and DC and T cell maturation induction. By inducing autophagy in tumor cells, we can indirectly stimulate multiple antitumor immune pathways, achieving potent tumor chemoimmunotherapy outcomes while ensuring biosafety considerations.

### Carbon-based nanomaterials

Carbon, as a common and abundant element, has a variety of isotopes, and different kinds of nanomaterials can be synthesized using different carbon elements, mainly fullerenes and their derivatives (fullerenol), carbon nanotubes (CNTs), nanodiamonds (NDs), and graphene oxide (GO) [99]. Carbon nanomaterials are more diverse, modifiable, and flexible than other common materials due to their small size, large specific surface area, good electrical and thermal conductivity, and other material characteristics [100]. Carbon-related nanomaterials are known to activate cellular autophagy mainly through mechanisms such as mitochondrial damage, increased ROS generation, and ER stress [101,102].

**Fig. 3.**
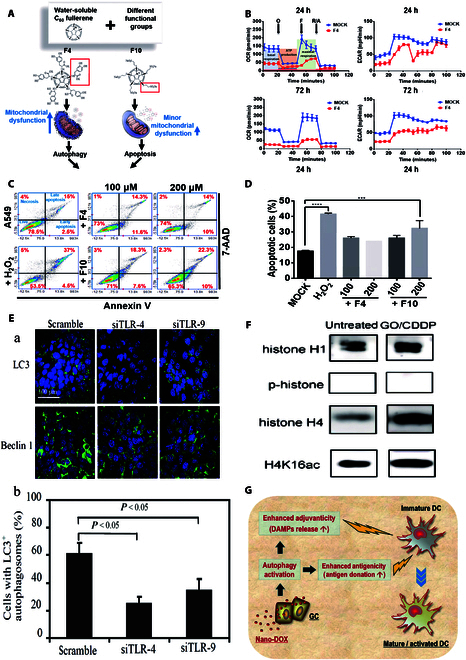
Carbon-based nanomaterials for the treatment of tumors by influencing autophagy. (A) Schematic of the different pathways by which the fullerene derivatives F4 and F10 destroy tumors [103]. (B) Effect of F4 fullerene derivatives (200 μM) on the mitochondrial function of A549 cells [103]. (C) Annexin-V/7-aminoactinomycin D (7-AAD) analysis of F4- or F10-treated A549 cells [103]. (D) Quantification of the sum of early apoptosis (lower right panel) and late apoptosis (upper right panel) phenotypes (C) [103]. (E) TLR-4/9 silencing reduced GO-induced autophagy. (a) GO-induced autophagy after TLR-4/9 silencing. (b) Quantification of cells with LC3 autophagosomes [111]. (F) Western blot analysis of proteins (~11 kDa and ~35 kDa) derived from the nuclear LC3-immunocomplex [110]. (G) Schematic of the activation of DCs via autophagy by nanoDOX [116]. Reproduced with permission [103]. Copyright 2019, American Chemical Society. Reproduced with permission [111]. Copyright 2014, Wiley. Reproduced with permission [110]. Copyright 2018, PubMed Central. Reproduced with permission [114]. Copyright 2019, Elsevier Ltd.

Fullerenes and their derivatives can also exert tumor-killing effects by modulating autophagy in tumor cells. Wen and colleagues [102] found that fullerene C60 induced autophagy in HeLa cells and MCF-7 cells at nontoxic concentrations, increasing their sensitivity to chemotherapeutic drugs, and is considered to have a potential adjuvant role in chemotherapy. Although the application of fullerenes to adjuvant chemotherapy has not been reported in the last decade, this approach provides an approach for modulating autophagy to sensitize cells to chemotherapy. Although this induction of autophagy does not apply to all types of fullerenes, Wong et al*.* [103] found that while both fullerene derivatives had autophagy-inducing effects, they had different roles in chemotherapy. The development of lung cancer A549 cells was shown to be suppressed by both fullerene derivatives, F4 and F10, although not by the same mechanism. F4 mostly caused mitochondrial damage and consequent autophagy, whereas F10 primarily caused ROS production and apoptosis (Fig. 3A to D). The mechanisms of autophagy induced by different derivatives are different. In summary, the mechanisms underlying the toxicity to normal cells and the killing of tumor cells by fullerenes are intricate, and research on the regulation of autophagy induced by fullerenes is currently quite limited. However, we anticipate that future studies will be able to investigate this topic in greater detail using particular cell and tumor models.

GO has been extensively applied in bioimaging, gene/drug delivery, cell detection, cell differentiation, and photothermal therapy due to its biocompatibility and ease of synthesis. GO-mediated autophagy has also been used in research in the field of tumor therapy, usually for chemotherapeutic sensitization [104–108]. Chen et al*.* [109] reported that GO can be used as a chemotherapeutic drug sensitizer. GO was found to promote autophagy in combination with the chemotherapeutic drug cisplatin, but this autophagy did not depend on the formation of autophagosomes or autophagic lysosomes but rather was achieved by increasing the translocation of LC3, not only to determine the sensitizing effect of GO on cisplatin chemotherapy but also to examine the GO-mediated autophagy mechanism. In addition, Lin et al*.* [110] investigated the mechanism by which GO in combination with cisplatin regulates the entry of LC3 into the nucleus and reported that this synergistic effect was observed in ovarian and cervical prostate cancer cells, in which LC3 and histones H1/H4 were able to comigrate into the nucleus, further elucidating the mechanism by which GO/chemotherapy drug cisplatin (CDDP) induces sensitization to chemotherapy (Fig. 3F). In addition to regulating the autophagy of tumor cells, GO also regulates the autophagy of immune cells such as TAMs in the TME. For example, in 2012, Chen et al*.* [111] reported that GO can activate autophagy in macrophages and Toll-like receptor 4/9 (TLR-4/9) to modulate inflammatory responses. Furthermore, in 2014, the group revealed that GO could activate TLRs and autophagy to fight tumors. GO (50 μg ml^−1^) activated TLR-4/9 and the downstream MyD88/TRAF6 signaling pathway to activate autophagy in the colon cancer cell line CT26. GO induces the activation of autophagy in various types of cells, which could play a role in sensitizing or immunomodulating tumors to chemotherapy immunomodulatory effects (Fig. 3E).

Because of their exceptional mechanical and optical qualities, high specific surface area, customizable surface structure, and chemical stability, NDs are frequently utilized in medication delivery, tissue scaffolds, and surgical implants [112]. Liu et al*.* [113] exploited the traceability of NDs through the ability of ubiquitin-coated NDs to selectively bind to LC3 and enter the selective autophagy pathway, which promotes fusion with lysosomes. The molecular mechanism by which NPs target human cells to induce selective autophagy has been explored, and understanding the molecular mechanism underlying the selective autophagy of nanomaterials will be helpful for the application of nanomaterials in biomedical fields such as drug delivery [113]. Li et al*.* [114] investigated the mechanism of autophagy activation-induced immunogenic activation of GCs and found that ND-loaded DOX was able to induce autophagy in GCs and release DAMPs, which in turn promoted DC development and controlled the immunological milieu that inhibits glioma (Fig. 3G).

The ability of carbon-based nanomaterials to disrupt cellular autophagy could be a mechanism of carbon NP cytotoxicity, and on the other hand, carbon-based nanomaterials could be exploited as an adjunct to tumor killing by increasing the susceptibility of tumor cells to chemotherapeutic drugs that cause autophagy-related cell death. Whether it is honey or poison depends on the modification and concentration of action of the carbon nanomaterials.

### Quantum dots

Semiconductor inorganic fluorescent nanocrystals with a diameter range of 1 to 10 nm are known as quantum dots (QDs), which are usually composed of a small number of atoms in groups II to IV or III to V of the periodic table of the elements to form quasizero-dimensional nanomaterials. QDs have unique excitation frequencies and broad emission spectrum properties and have better anti-interference and target binding abilities than traditional fluorescent dyes; therefore, QDs are frequently employed as fluorescent probes in fixed-cell bioimaging studies as contrast agents for medication administration [115]. By comparing the autophagy results and cytotoxicity induced by QDs of different sizes in human bone marrow mesenchymal stem cells, Seleverstov et al*.* [116] discovered that nanomaterials can induce size-dependent cellular autophagy effects, and a series of subsequent studies demonstrated that a variety of QDs can induce autophagy. Later, Stern et al*.* [117] reported that autophagy could be induced in pig kidney cells by the same QDs with identical particle sizes made of various components, and the degree of autophagy was directly correlated with the degree of cytotoxicity caused by the QDs.

**Fig. 4.**
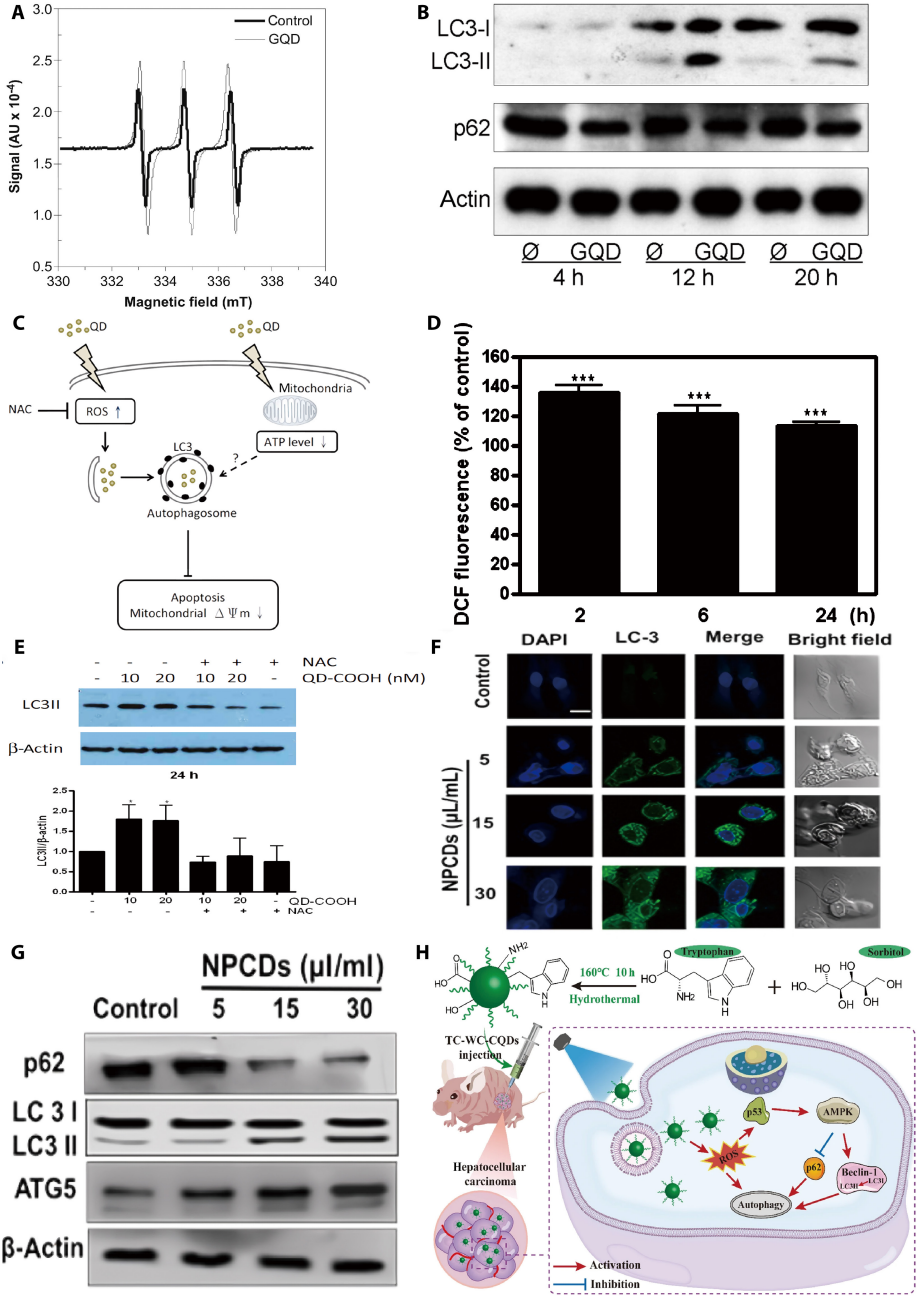
QDs for the treatment of tumors by influencing autophagy. (A) ROS production by photoexcited GQDs under cell-free conditions. Enhanced permeability and retention effect (EPR) analysis of singlet oxygen production in light-irradiated (470 nm, 1 W, 30 min) GQDs and control solution [arbitrary units (AU)] [118]. (B) Role of autophagy in the intracellular localization and phototoxicity of GQD. Immunoblot analysis of LC3 conversion and p62 levels in U251 cells incubated with control solution or GQD (200 μg/ml) and exposed to blue light (470 nm, 1 W) for 10 min [118]. (C) Model of oxidative stress and autophagy induced by QDs in RAG cells. NAC reduces intracellular ROS levels and decreases QD-induced autophagy but enhances QD-induced cell death. ROS play an essential role in the regulation of QD-induced autophagy, which subsequently enhances cell survival. ΔΨ, mitochondrial membrane potential [119]. (D) ROS production in RAG cells treated with 20 nM QDs for 2, 6, or 24 h [119]. (E) Effect of NAC on QD-induced autophagy. LC3-II expression in 10 and 20 nM QD-treated cells at 24 h with or without NAC (5 mM) pretreatment [119]. (F) Immunocytochemistry of the prominent autophagy marker LC3 after treatment with NPCDs evaluated via confocal microscopy [121]. (G) Western blot analysis of various autophagy markers (LC3, ATG-5, and p62) [121]. (H) Schematic diagram of the TC-WS-CQD synthetic diagnostic treatment for hepatocellular carcinoma [122]. Reproduced with permission [118]. Copyright 2012, Elsevier. Reproduced with permission [119]. Copyright 2013, American Chemical Society. Reproduced with permission [121]. Copyright 2020, PubMed Central. Reproduced with permission [122]. Copyright 2022, BioMed Central.

Graphene QDs (GQDs) are a zero-dimensional graphene material characterized by graphite planes with atomic-level thickness (less than 2 nm thick) and lateral dimensions typically less than 10 nm. Given their exceptional characteristics, including their strong quantum confinement effect, chemical stability, low toxicity, and stable photoluminescence (PL), GQDs are being investigated as innovative materials for applications in biology, photovoltaics, energy, and the environment. Markovic et al*.* [118] synthesized and prepared GQDs and incubated them with human GCs (U251) and found that under blue light irradiation with an excitation wavelength of 470 nm and a power of 1 W, QDs stimulated cells to produce reactive oxygen species and killed tumor cells through oxidative stress. QDs stimulate a bleaching effect in cells. This stimulates cytotoxicity of the mitochondria in the cells, increases the level of oxidative stress in the cells, and increases intracellular ROS accumulation. The accumulation of ROS induced cell autophagy and killed U251 GCs. With increasing stimulation time, the expression of the autophagy signature protein LC-3B increased, and the autophagy substrate P62 was consumed, which partially eliminated the photodynamic cytotoxicity induced by GQDs [118]. It was concluded that QDs trigger autophagy by inducing intracellular oxidative stress (Fig. 4A and B).

Among cadmium-containing QDs, researchers chose mouse renal adenocarcinoma (RAG) cells, and the findings demonstrated that within 6 h of treatment, GQD-treated RAG cells significantly increased their levels of ROS and cell autophagy, and cell apoptosis ensued within the subsequent 24 h [119]. The researchers found that the oxidative stress induced by GQDs exhibited a dual regulatory effect on the autophagy induced by immune cells; on the one hand, GQDs stimulated the mitochondria to produce ROS, induced autophagy, and exhibited a proinflammatory response; on the other hand, GQDs only affected the mitochondria and did not induce ER stress, and the *N*-acetylcysteine (NAC) reduced the level of intracellular ROS and inhibited cellular autophagy; similarly, 3-MA promoted cell death in the QD-treated group, thereby promoting tolerogenic DC differentiation and suppressing the T helper 1 (T_H_1)-type immune response. Purpose QD-mediated autophagy is mainly accomplished through ROS regulation, but autophagy can be affected by a variety of factors, such as QD composition or cell type (Fig. 4C to F) [119].

Carbon dots (CDs) are the intersection of semiconductor QDs and carbon materials and have superior optical properties (e.g., PL, tunable emission wavelengths, and broadband absorption) to traditional QDs. Furthermore, CDs have an abundance of surface functional groups (such as carboxyl, carbonyl, and hydroxyl groups) that contribute to their good water solubility and ease of surface modification and coupling. These properties greatly ease the use of CDs in bioimaging and detection applications. CDs are typically selected as raw materials for the manufacture of nanocarriers when their particle size is less than 10 nm. Taking advantage of this property of the material, our group has developed a multifunctional dual colorimetric–polyacrylamide–quaternary ammonium chitosan–carbon–quantum dot (CQD)–phenol red hydrogel for real-time monitoring of wound healing through the observation of changes in wound pH and fluorescence signals [120]. Recently, researchers have shown that CD can penetrate the blood–brain barrier (BBB) and induce tumor cell death via photothermal effects, while CD also induces mitochondrial stress and induces apoptosis by mediating cellular autophagy. Bajpai et al*.* [121] synthesized nitrogen–phosphorus-doped CDs (NPCDs) for melanoma treatment using heat treatment, and the synthesized substance suppressed melanoma development in a dose-dependent manner, according to the experimental data. The results of the present study demonstrated that the synthetic material inhibited the progression of melanoma in a dose-dependent manner, activated cellular autophagy by up-regulating the expression levels of the ATG-5 and LC3-II proteins and down-regulating the level of p62, and induced apoptosis by promoting the synthesis of ROS and disrupting the function of mitochondria. Through the combined effect of these multiple aspects, NPCDs showed excellent antitumor activity in the treatment of melanoma, and the material was also capable of bioimaging of phagocytosed cells (Fig. 4G) [121]. Similarly, the tryptophan–sorbitol CQDs (TC-WS-CQDs) designed by Wang et al*.* [122] were able to undergo a large number of redox reactions. Because they are capable of generating a large amount of ROS, they induce hepatocellular carcinoma (HCC) autophagy through the p53-AMPK pathway, and their fluorescence imaging ability makes them comprehensive reagents for diagnosis and treatment, which helps in the early detection of hepatocellular carcinoma (Fig. 4H). In conclusion, QD-mediated autophagy is mainly accomplished through ROS regulation, but autophagy can be affected by a variety of factors, such as QD composition or cell type.

As a commonly used raw material for drug carriers, QDs can also be applied in chemotherapeutic drug delivery, and QDs are commonly paired with 5-fluorouracil (5-FU). In previous studies, cellular autophagy was shown to enhance tumor resistance to 5-FU and reduce drug efficacy, while the development and use of QD nanocarriers targeted 5-FU and autophagy inhibitors to tumor cells; thus, when 5-FU has antitumor efficacy, autophagy inhibitors inhibit cellular autophagy, reduce cellular resistance, increase tumor sensitivity to radiotherapy, and improve therapeutic efficiency. In a related study on lung cancer treatment, researchers successfully developed the Ag_2_SQD nanomaterial, and PEG was applied to the surface of the material to improve its biocompatibility and blood circulation half-life. At the same time, the material was labeled with cetuximab (Cet) to enable near-infrared (NIR) imaging and detection of epidermal growth factor receptor (EGFR)-expressing tumor cells with high imaging efficiency [123]. With the assistance of Cet, QDs can be used to selectively deliver 5-FU, which targets highly EGFR-expressing cells, realizing precise drug transport and enhancing cell death associated with apoptosis. Moreover, related experiments demonstrated that compared to direct drug delivery, the use of material-assisted delivery of 5-FU significantly overcame cellular drug resistance and improved therapeutic efficacy, which has been attributed to the synergistic effect of enhanced receptor-mediated NP uptake by Ag_2_SQDs, which can bind to inhibited autophagy, even without the addition of autophagy inhibitory factors [123].

In addition to its modulatory effect on autophagy in tumor cells, it also modulates autophagy in immune cells. Tomić et al*.* [124] investigated the mechanism of QD-induced autophagy modulation in immune cells and reported that nontoxic doses of GQDs promoted monocyte-derived tolerogenic DCs that are detrimental to antitumor therapy through activation of autophagic streams in DC cells, increased transcription of ATGs, and increased ATG function. Clarification of this mechanism may enhance the therapeutic application of QDs in oncology.

In summary, QD-regulated autophagy plays an important role in cell fate determination. Like other nanomaterials, QD-induced moderate autophagy activation is a defense/survival strategy against nanotoxicity [119]. On the other hand, excessive autophagy induced by QDs promotes cell fate decisions [125].

### Silicon-based nanomaterials

Nanomaterials based on silica are widely employed in medication delivery, bioimaging, and disease detection due to their excellent electrical and mechanical properties, high specific surface area, easily modifiable surfaces, and good biocompatibility [126–128]. Hollow porous silica structures can be used to carry different drugs (e.g., chemotherapeutic agents and autophagy modulators) to treat tumors, and their modifiable surfaces can be modified to exert anticancer effects through polymers (e.g., dopamine). Moreover, silica-based nanomaterials also have autophagy-modulating effects.

**Fig. 5.**
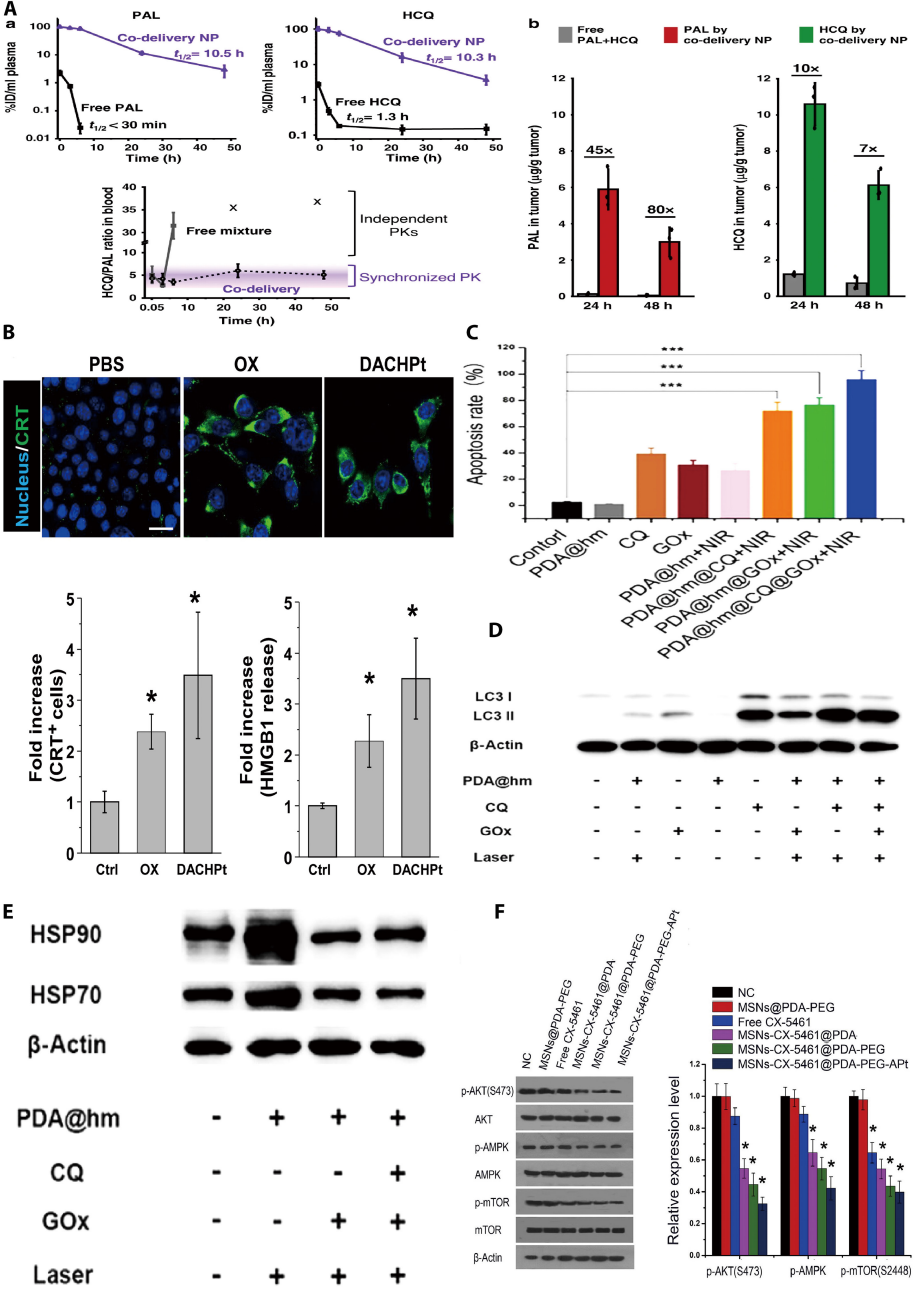
Silica-based nanomaterials for the treatment of tumors by influencing autophagy. (A) Mice received a single intravenous injection of co-delivery NP (with the particle dose of 210 mg/kg) or free PAL/HCQ pair. (a) Evaluation of PK profile and PAL/HCQ ratio in plasma. Plasma was collected 0.083, 3, 6, 24, and 48 h post intravenous injection (n = 3 animals included in each data point). The plasma content of PAL and HCQ was determined by UPLC-MS and expressed as percentage of total injected dose (% ID) per mL of plasma. (b) PAL/HCQ content at the tumor site. In a separate experiment, mice were sacrificed at 24 or 48 h post intravenous injection (n = 3 animals included in each data point). PAL/HCQ content in tumor site was determined by UPLC-MS, and comparison was made between co-delivery NP and direct administration of free PAL/HCQ mixture [129]. (B) 1,2-Diaminocyclohexane-platinum(II) (DACHPt) induces immunogenic cell death (ICD). Upper panel: Confocal microscopy image showing CRTs on the surface of KPC cells after treatment with oxaliplatin or DACHPt (500 μM) for 24 h. Green, CRTs; blue, nuclei. Lower panel: CRT expression was assessed by flow cytometry (left panel), and HMGB1 release was measured by enzyme linked immunosorbent assay (ELISA) (right panel) in KPC cells treated with oxaliplatin or DACHPt (500 μM) for 24 h [130]. (C) Cell cytotoxicity was augmented by mild PTT in combination with the glucose consumer GOx and the autophagy inhibitor CQ in HepG-2 cells [131]. (D) Relative LC3-I and LC3-II expression in HepG-2 cells after different treatments. β-Actin was used as an internal control [131]. (E) Representative expression of HSP90 and HSP70 after different treatments determined by Western blot analysis [131]. (F) HeLa cells were subjected to the indicated treatments for 6 h, after which Western blotting was performed. Densitometry of the Western blot bands was performed [133]. Reproduced with permission [129]. Copyright 2020, PubMed Central. Reproduced with permission [132]. Copyright 2021, Wiley. Reproduced with permission [131]. Copyright 2020, PubMed Central. Reproduced with permission [133]. Copyright 2018, Elsevier.

For example, Shen et al*.* [128] used hollow mesoporous silica nanospheres to encapsulate bortezomib, a drug delivery system that activates autophagy in tumor cells and increases anticancer activity by approximately 1.5-fold compared to that of free bortezomib (BTZ). Numerous research teams have treated pancreatic cancer by using the medication delivery capabilities of silica-based NPs. Ji et al*.* [129] used mesoporous silica in conjunction with lipid membrane encapsulation to codeliver precise ratios of the autophagy inhibitor HCQ and the CDK4/6 inhibitor palbociclib (PAL). This allowed the medications to accumulate more fully inside the tumor, and the proportional codelivery of the nanocarriers inhibited autophagy compared to that in a variety of control groups that included free pairs of drugs of the nanocarriers inhibited autophagy-induced apoptosis, resulting in a potent pancreatic cancer therapeutic effect (Fig. 5A).

In addition, when liposome membrane-encapsulated mesoporous silica NPs are used for drug delivery for the treatment of pancreatic cancer, the pH of the loaded drug also affects the therapeutic effect. For example, the mesoporous silica NPs prepared by Liu et al*.* [130] encapsulated a lipid bilayer cell membrane called a silica colloid, and the loaded drug was weakly alkaline, which could neutralize the acidic pH of the lysosomes of pancreatic ductal adenocarcinoma (PDAC) cells and inhibit the occurrence of cellular autophagy. PDAC cells can inhibit the occurrence of cellular autophagy and induce cellular immunocompromised death (ICD), which could have a killing effect on the injection site through the tumor immune system on the opposite side of the tumor (Fig. 5B) [130].

In addition to the modification of silica NPs using lipid membranes, polydopamine is also frequently used for the modification of silica NPs. Dopamine-encapsulated mesoporous silica was created by Shao et al*.* [131] for photothermal treatment (PTT) and in vivo photoacoustic demonstration. The effectiveness of PTT for tumor cells increased with the addition of autophagy inhibitors. Polydopamine is not only a photothermal material but also a gatekeeper for drug release, enabling targeted intracellular release of drugs to reach and accumulate in specific organelles (Fig. 5C to E) [131]. The dopamine-encapsulated NPs constructed by Ding et al*.* [132] were able to shed within lysosomes, target the release of drugs, cause lysosomal damage, and prevent autophagy from occurring, thereby increasing the effectiveness of tumor therapy. Unlike Ding et al*.*, who reported lysosomal damage to inhibit autophagy, the polydopamine mesoporous silica nanomaterials constructed by Duo et al*.* [133] were equipped with a ribosomal RNA (rRNA) synthesis inhibitor (CX-5461), which induced death-promoting autophagy (Fig. 5F). Polydopamine surface-modified AS-1411 was able to induce nuclear aggregation, whereas polydopamine (PDA) prevented CX-5461 from leaking before reaching the nucleus, thereby enhancing tumor therapy by inducing lethal autophagy [133].

### Liposomes

Liposomes are an important class of lipid-based nanomaterials that were first discovered by Bangham et al*.* in 1964 [134]; they consist of single or multiple concentric phospholipid lipid bilayers with good biocompatibility, natural degradability, cell membrane fusion, and high encapsulation [135] and are widely used in biomedical fields such as drug delivery, biosensors, and nutrient delivery [136–138]. Furthermore, by interacting with mononuclear phagocytes, researchers have discovered that liposomes can cross the BBB and be eliminated from the bloodstream. Additionally, liposomes have been shown to improve intracellular drug accumulation and in vivo circulation time, which offers promising therapeutic outcomes for the treatment of tumors. These findings are further supported by hydrophilic polymers such as poly(ethylene glycol) (PEG). Along with a number of oncology therapeutics currently undergoing clinical development trials, the U.S. FDA has approved a number of liposomal medications for experimental clinical applications. These include Myocet for metastatic breast cancer, DaunoXome for Karposi sarcoma, Marqibo for acute lymphoblastic leukemia, Onivyde for pancreatic cancer, and Doxil/Caelyx, among others [139,140].

**Fig. 6.**
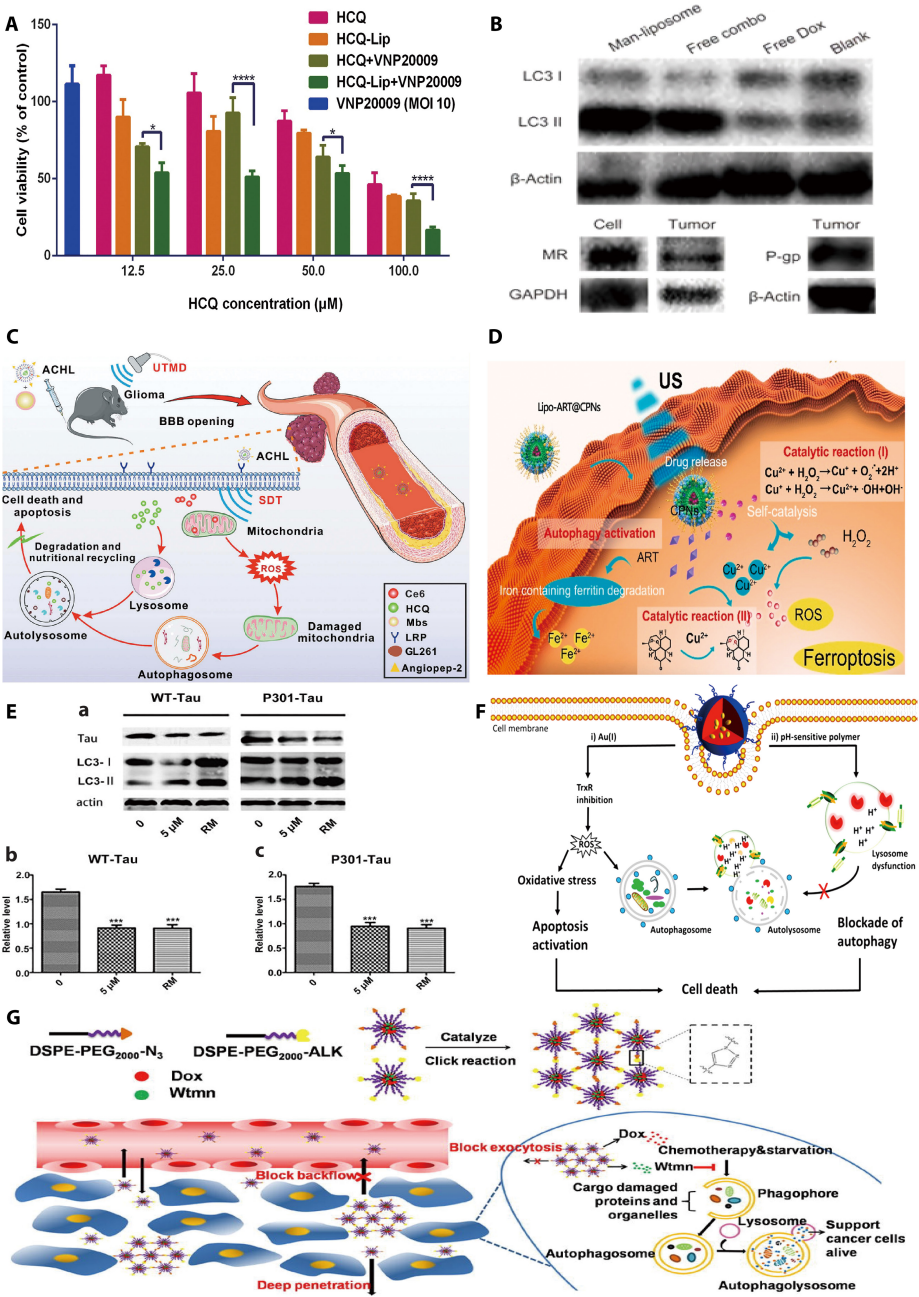
Liposomes for the treatment of tumors by influencing autophagy. (A) Methylthiazolyldiphenyl-tetrazolium bromide (MTT) assay of B16F10 cells treated with HCQ or HCQ-Lip (50 μM) for 15 min and then infected with VNP20009 at a multiplicity of infection (MOI) of 10 for 3 h [141]. (B) The MR, P-gp expression on HCT8/ADR tumors and cells, and the expression of LC3 of HCT8/ADR cells after different treatments (the DOX-resistant human colon cancer cell line, HCT8/ADR) [143]. (C) Manipulation of mitophagy by “All-in-One” nanosensitizer augments sonodynamic glioma therapy [148]. (D) Engineering dual catalytic nanomedicine for autophagy-augmented and ferroptosis-involved cancer nanotherapy [149]. (E) PEG-ceramide nanomicelles promoted autophagy and degraded tau protein in N2a cells. (a) Western blotting of the LC3-I, LC3-II, and tau proteins in N2a cells treated with PEG-ceramide nanomicelles. RM (rapamycin, 5 μM) was used as a positive control. (b and c) Graph of protein levels normalized to β-actin levels. All other groups were compared to the untreated control group by one-way analysis of variance with Dunnett’s post hoc test [151]. (F) pH-sensitive polymeric NPs with gold (I) compound payloads synergistically induce cancer cell death through modulation of autophagy [153]. (G) Size-adjustable micelles coloaded with a chemotherapeutic agent and an autophagy inhibitor for enhancing cancer treatment via increased tumor retention [156]. Reproduced with permission [141]. Copyright 2017, PubMed Central. Reproduced with permission [143]. Copyright 2017, Nature Portfolio. Reproduced with permission [148]. Copyright 2020, PubMed Central. Reproduced with permission [149]. Copyright 2022, Elsevier. Reproduced with permission [151]. Copyright 2020, PubMed Central. Reproduced with permission [153]. Copyright 2015, American Chemical Society. Reproduced with permission [156]. Copyright 2019, Elsevier.

The use of liposomal encapsulation of autophagy-modulating drugs such as HCQ can prolong the circulation time of the drug. Wang et al*.* [141] discovered that the drug circulation period was extended by liposome encapsulation, which enhanced the effect of HCQ on autophagy and enhanced the therapeutic efficacy of Salmonella on invasive melanoma (Fig. 6A). In addition to encapsulating autophagy-modulating drugs alone, liposomes can codeliver autophagy-modulating drugs or genes with therapeutic agents. In an earlier study, our group found that liposomal codelivery of salinomycin and CQ could exert a synergistic anticancer effect in vitro through the inhibition of autophagy [142]. Kang et al*.* [143] designed a mannosylated liposomal nanocarrier for the codelivery of DOX and dihydroartemisinin (DHA) for the treatment of multidrug-resistant colon cancer, and the codelivered group showed a longer time of release, stronger autophagy inhibition, and optimal tumor growth than did the free drug group (Fig. 6B). The study also revealed that this combination did not increase the toxicity of the drug. In addition to drug delivery, liposomes are also commonly used nonviral gene delivery systems, and more importantly, liposomes are capable of codelivering genes and drugs [144]. Some studies have shown that the codelivery of tumor therapeutic genes or miRNAs with chemotherapeutic drugs can enhance the therapeutic effect of tumor therapy [143], but to our knowledge, no evidence of the codelivery of autophagy-related gene siRNAs with chemotherapeutic medicines to improve the therapeutic benefit has been reported.

In addition to their superior drug encapsulation and delivery properties, the surface modifications of liposomes allow them to target cells specifically for the administration of autophagy-regulating medicines, thus regulating autophagy more precisely. Zheng et al*.* [146] constructed a “three birds, one stone” delivery strategy by modifying liposomes using a7 nicotinic acetylcholine receptor (nAChR)-linked peptides to increase delivery to vascular endothelial cells, GCs, and TAMs. These altered liposomes dramatically increased tumor cell autophagy, which enhanced immunogenic tumor cell death and enhanced antitumor effects. The targeting strategy for autophagy modulation varies from tumor to tumor. For example, the main reason for chemoresistance in pancreatic cancer is the dense structure of mesenchymal cells [e.g., cancer-associated fibroblasts (CAFs)] surrounding pancreatic cancer cells, which makes penetration of chemotherapeutic drugs difficult. Selecting integrins for liposome modification can increase the targeting ability to mesenchymal cells such as CAFs, and autophagy-inhibiting drugs can reduce the formation of CAFs and prevent mesenchymal fibrosis, thus enhancing the penetration of drugs and exerting anticancer effects [147]. By integrating the above strategies, we also designed an “all-in-one” liposome synergistic drug delivery strategy that utilizes vascular endothelial inhibitor 2 peptide-modified liposomes encapsulated with the sonication activator chlorin e6 (Ce6) and the autophagy inhibitor HCQ to increase chemotherapeutic drug penetration into the BBB via ultrasound-targeted microbubble disruption (UTMD) under the action of ultrasound. Autophagic vesicles cannot be disrupted by the autophagy inhibitor HCQ, thereby amplifying the oxidative stress damage caused by intracellular ROS, enhancing tumor inhibition, and prolonging the survival of mice (Fig. 6C) [148]. According to the above mechanism, dual catalytic strategy-induced and autophagy-enhanced iron mutations could promote and direct cancer cell death.

In addition, the efficacy of tumor therapy can also be achieved by simultaneously increasing endogenous hydrogen peroxide production and regulating autophagy in the microenvironment. Li et al*.* [149] constructed a multifunctional liposomal nanosystem that encapsulated and codelivered copper peroxide nanoparticles (CPNs) and artemisinin (ART) simultaneously. Specifically, copper peroxide nanodots and ART were induced to be released from the liposomal nanosystem to the vicinity of the tumor site by ultrasonic irradiation. On the one hand, increased intracellular oxidative damage and lipid peroxide accumulation were induced by the endogenous production of ·OH and ROS free radicals generated through a dual Cu-based catalytic strategy. On the other hand, intracellular iron levels were increased through ferritin degradation due to the strong autophagy-inducing ability of ART (Fig. 6D) [149].

Furthermore, in addition to their role as carriers for drug delivery, liposomes can directly impact cellular autophagy posttreatment. Previous research conducted by our group focused on examining the regulation of autophagy in DC cells by liposomes containing various anions and cations [150]. The cytotoxic nature of cationic liposomes (CLs) prevents them from being used as nonviral vectors. CLs led to an increase in lysosomal pH within DCs. This alteration could decrease antigen degradation, increase the surface expression of MHC-I peptide complexes, facilitate cross-presentation of antigens, and stimulate the specific activation and proliferation of CD8^+^ T cells. Conversely, anionic liposomes (ALs) did not significantly impact lysosomal pH or antigen processing. In summary, CLs primarily inhibit cellular autophagy by inducing lysosomal alkalinization in DC cells [150]. This mechanism can be harnessed for antitumor immunomodulation through autophagy manipulation, thereby enhancing the efficacy of antitumor vaccines.

Lipid-based micelles are another promising type of lipid-based nanocarrier for tumor therapy. Like liposomes, micelles are formed by the self-assembly of amphiphilic polymers in a simple manner, and lipid-based micelles are capable of autophagy-associated drug delivery, long cycling, and surface modification. The difference lies in the ability of the micelles themselves to modulate tumor autophagy.

We used PEG-ceramide micelles on N2a cells and found that these micelles induced autophagy and increased the degradation of the Tau protein in N2a cells (Fig. 6E) [152]. The self-assembled autophagy-inducing micelles constructed by Wang et al*.* [153] have autophagy-inducing effects, and the autophagy level of MCF-7 cells can be further increased by altering the autophagy-inducing peptide Bec1 on the surface of these micelles, which would boost the therapeutic effect of tumor therapy. Poly (β-amino ester)-based pH-sensitive micellar NPs self-assemble and stimulate cellular autophagy, which enhances the therapeutic efficacy of tumor therapy, as reported by Lin et al*.* [153]. The resulting gold(I)-loaded NPs [Au(I)⊂NPs] encapsulated gold(I) compounds [Au(I)] in the hydrophobic domains of the NPs, which entered the cells via endocytosis, where they gathered and were eliminated in acidic lysosomes. In addition, Au(I) molecules can be triggered and released due to tertiary amine protonation of poly(β-amino ester). This inhibits TrxR activity and thus indirectly increases intracellular ROS, which not only enhances oxidative stress but also induces autophagy (Fig. 6F) [153]. Thus, Au(I) micellar nanocarriers that synergistically induce cell death by modulating autophagy have a synergistic role in killing cancer cells.

Like liposomes, micelles can regulate autophagy through drug or gene codelivery. Micelles codeliver chemotherapeutic drugs with autophagy inhibitors (e.g., CQ) [154] or siRNAs targeting autophagy-related genes (e.g., ATG7) [155], which can enhance the sensitivity to chemotherapeutic drugs. Most of these codelivery strategies use autophagy inhibitors to modulate the protective autophagy initiated by tumor cells in the face of a lethal threat, thereby improving the efficacy of chemotherapy, photothermal therapy, and other tumor treatment strategies. To codeliver the chemotherapeutic drug DOX and the autophagy inhibitor wortmannin, Rao et al*.* [156] used Cu(I)-catalyzed click chemistry-triggered aggregation of azide/alkane-modified micelles [based on Cu(I)-catalyzed click chemistry-triggered aggregation of azide/alkane-modified micelles]. This method increases the therapeutic efficacy of chemotherapeutic drugs in melanoma and breast cancer (Fig. 6G).

Since protective autophagy in tumor cells reduces the efficacy of photothermal therapy, photothermal therapeutic micelles containing autophagy-modulating drugs were developed. Chen et al*.* [157] constructed an in situ self-assembled micelle containing the autophagy inhibitor CQ and the cellular immune-death inducer IR780. CQ blocked protective autophagy in tumor cells, thereby improving the efficacy of photothermal therapy and immunotherapy. Autophagy modulation also affects tumor sensitivity to radiotherapy, and to the best of our knowledge, we have not seen studies using micelles to deliver autophagy-modulating drugs for radiotherapy sensitization, which could be explored in future studies.

In addition to loading drugs that directly modulate autophagy, micelles can also be loaded with drugs that affect cellular biological processes to modulate autophagy. For example, overactivated mitophagy can deplete ATP to activate energy-dependent cellular autophagy [158]. Zhu et al*.* [159] took advantage of this property and constructed drug-loaded micelles targeting mitochondria, which can overactivate mitophagy to activate cellular autophagy and ultimately lead to cell death. At the same time, the micelles accumulated in the cell mitochondria can further destroy the mitochondria and kill the cells through photothermal or photodynamic effects.

In summary, lipid-based nanomaterials, as nanodelivery platforms with broad clinical applications, mainly rely on the codelivery of autophagy modulators to enhance the anticancer effects of traditional therapeutic modalities, and although more breakthroughs have been achieved in recent years, there are still some niche areas that need to be further explored.

## Conclusion

**Fig. 7.**
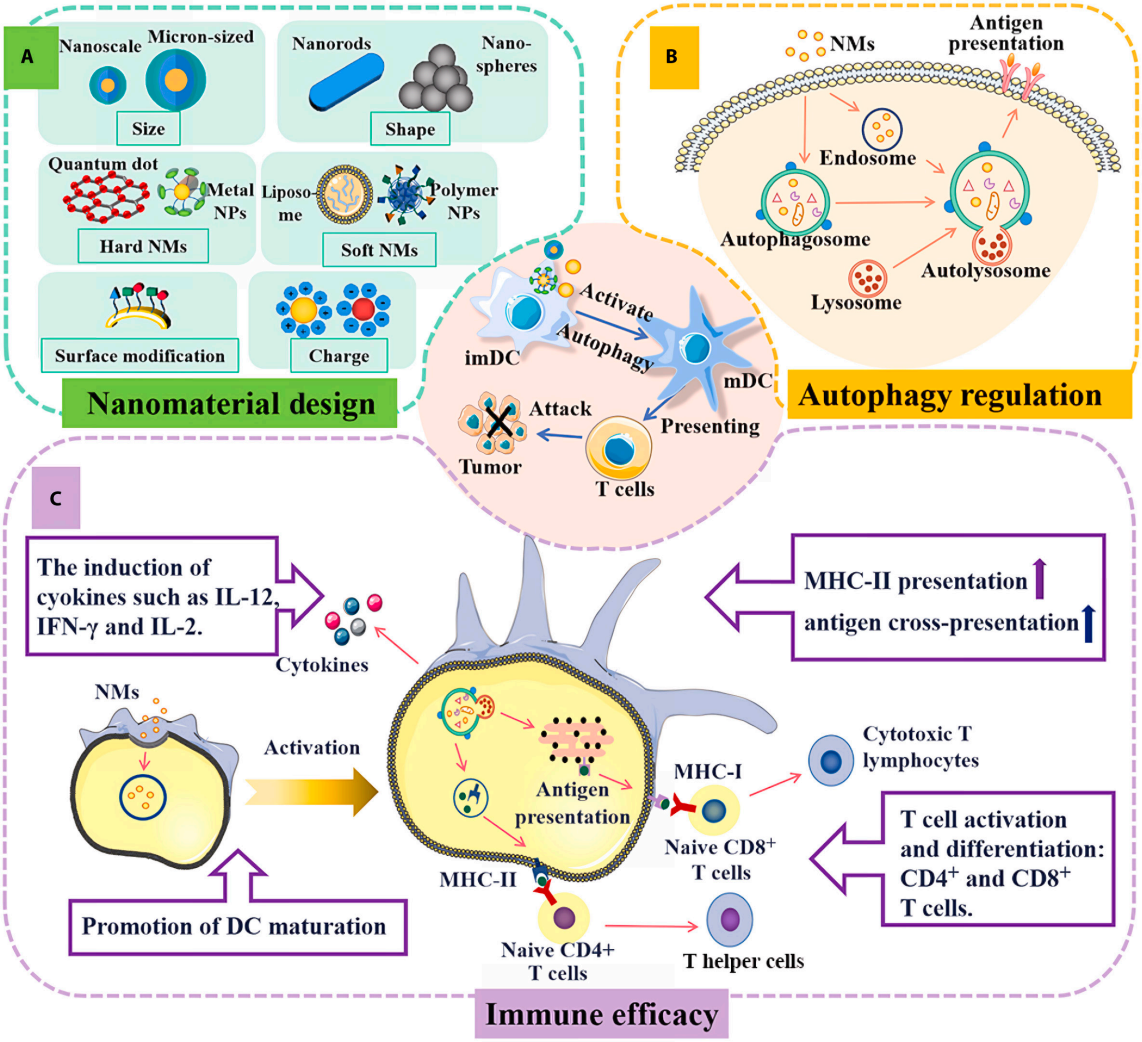
Autophagy-based nanomaterials enable antitumor resistance [160]. The exploration of DC activation by autophagy-regulating nanomaterials in tumor immunotherapy. The following 3 aspects are described: (A) The physicochemical properties of nanomaterials, including materials, shape, size, surface modification, and charge, are analyzed and adjusted to achieve optimal DC autophagy regulation. (B) The mechanism and pathway of autophagy regulation by nanomaterials are summarized to achieve the objective of activating DCs. (C) The effects of nanomaterials on DC autophagy in enhancing the antitumor immune response are discussed from several perspectives, including promoting DC maturation, facilitating antigen presentation, boosting the release of immunostimulating cytokines, activating T cells, and adjusting their differentiation.

This review synthesizes the amalgamation of nanomaterials with oncological therapeutic strategies, elucidating the evolution of autophagy modulated by these materials within the realm of tumor immunotherapy. Given the ambivalent role of autophagy in tumorigenesis, its modulation, by either promotion or inhibition, can be strategically employed to counteract malignancy. The integration of autophagy modulation with conventional and innovative therapeutics has yielded commendable success in tumor management. This manuscript describes 5 classes of nanomaterials, namely, carbon-based NPs, QDs, silicon-based nanomaterials, metallic nanomaterials, and liposomes, and provides an in-depth analysis of their autophagy-regulating mechanisms (Fig. 7).

Among metallic nanomaterials, this review introduces a spectrum of materials, such as gold, silver, copper, iron, and zinc, detailing their intrinsic properties and their capacity to regulate autophagy. These materials exert their influence predominantly through direct interactions with autophagic cells, DCs, and tumor cells, modulating lysosomal pH, membrane permeability, and autophagic flux (Table 2). Additionally, certain materials are capable of triggering oxidative stress by modulating the release of ROS from the mitochondrial membrane. Carbon-based nanomaterials and QDs similarly regulate autophagy through their inherent material properties, while silicon-based nanomaterials and liposomes are predominantly utilized as drug delivery systems to modulate cellular autophagy in conjunction with encapsulated therapeutics.

**Table 2.** The mechanism by which nanomaterials regulate autophagy in cancers

Items	Nanomaterials	Autophagic markers	Other markers	Effects	Ref.
Cancer cell	HSA-C5 complex NPs	LC3B-II↑ Beclin 1↑	TCL1↓ Bcl-xL↓ AKT↓ p-AKT↓ p-Bad↓ Bcl-2↓ Caspase-10↓	Inducing autophagy, apoptosis, and inhibiting the PI3K–Akt signaling pathway.	[161]
	Nano-ZnO	LC3↑	P53mRNA↑, Caspase-3↑	Inducing autophagy, up-regulating p53 gene, facilitating the apoptosis of liver cancer cells.	[162]
	GCD@DOX	LC3-II/I↑ P62↑	/	Inducing autophagy, evade chemoresistance and doxorubicin toxicity	[163]
	Autophagy sensitive nanoparticle	LC3-II/I↑	HMGB1 release↑	Transforming autophagy to overactivated condition, leading tumor cells to autophagic death and enhancing subsequent tumor antigen processing of the dying cells	[164]
	Au@Cu_2−*x*_Se NPs	LC3-II↑ SQSTM1/P62↑	Rad51 ubiquitination↑	Inhibiting protective autophagy and DNA repair, suppressing the growth of orthotopic glioblastoma	[99]
	ICGCQ@RCm NPs	LC3-II↑ LC3-I↑ P62↑	Hsp70↑ CRT expression↑	Reducing the thermal tolerance of tumor cells to sensitize ICG-mediated photothermal therapy, inducing immunogenic cell death, suppressing tumor metastasis, and prolonging the survival time of tumor-bearing mice	[165]
	CD-Ce6-3BP NPs	LC3-II/I↑ P62↓	ATP↓ GADPH↓	Activating autophagy, promoting cell apoptosis, inhibiting cell proliferation, and promoting tumor regression	[166]
DC	α-Fe_2_O_3_	P62↑	/	Delivering antigens to autophagosomes in dendritic cells, which then presented the antigens to T cells through autophagy	[167]
	NP-B-OVA nanoactivators	LC3-II↑ P62↑	/	Induction of autophagy leads to efficient antigen expression in dendritic cells and generation of antigen-specific T cells	[168]
	Superparamagnetic iron oxide nanoparticle	LC3-II↑	/	Antigen delivered in cytoplasm induced by positive charged particles is beneficial for antigen cross-presentation and T cell activation	[169]
	N-Alkylpolyethylenimine coated iron oxide nanoparticles	LC3-II↑ P62↑	CD80^+^↑	Inducing autophagy and DC maturation	[170]
	Doxorubicin–polyglycerol–nanodiamond composites	LC3-II↑ Beclin 1↑ P62↑	MHC-II↑ CD80↑ CD86↑ CD11c↑	Inducing autophagy and enhancing the activation of DCs	[140]
	Graphene oxide	LC3-II↑ Beclin 1↑	MHC-II↑ CD86↑	Promoting the activation of APCs and antigen cross-presentation	[171]
Macrophage	Gold nanoparticle	LC3-II↑ Beclin 1↓ P62↑	/	Suppression of macrophages polarization to M2 phenotype by autophagy blockade	[95]
	DNA nanodevice	LC3-II↑ P62↑	Surface MHC-I-bound OVA_257–264_↑	DNA nanodevices target macrophages to modulate their cross-present antigen ability	[172]

Despite the significant progress in nanomedicine and the established efficacy of autophagy regulation by nanomaterials, further investigations are needed. Initially, the fabrication of novel NPs must be refined to enhance their efficiency in antitumor therapies. A thorough exploration of the physicochemical characteristics of NPs is imperative to maximize their benefits and minimize their drawbacks, including their size, rigidity, composition, and electrical charge. Subsequently, the specificity of autophagy induction by NPs warrants further investigation in both experimental and clinical cancer contexts to achieve a precise understanding of their effects and to circumvent potential side effects. Furthermore, NP-mediated autophagy signaling pathways need to be evaluated to elucidate the reciprocal influence between the TME and NPs. While NP-induced autophagy typically exhibits a pro-cell death effect, certain NPs may trigger pro-survival autophagy in specific cell types. Therefore, delineating the precise molecular mechanisms and downstream signaling pathways altered by NP-induced autophagy within tumor cells is essential for optimizing NP application in tumor therapy. Additionally, the exploration of synergistic combinations of autophagy regulation with other antitumor strategies, both traditional and contemporary, is crucial for enhancing antitumor efficacy. Finally, emerging novel technologies should be harnessed to assess the utility of NPs in regulating autophagy within the TME. The dualistic effects of autophagy underscore the potential of NP-induced autophagy in inhibiting tumor cell survival, directly addressing therapeutic demands. However, under certain conditions, the inhibition of NP-induced autophagy by autophagy inhibitors may be necessary to achieve antitumor effects. Although nanomaterials have been shown to regulate autophagy and contribute to tumor treatment, the development of novel NPs with a significant therapeutic impact on tumor immunotherapy and a deeper understanding of the mechanisms underlying nanomaterial-induced autophagy regulation demand ongoing research.

## Data Availability

No data were used for the research described in the article.
